# Nanoparticle Detection in Biology and Medicine: A Review

**DOI:** 10.3390/bios15120809

**Published:** 2025-12-11

**Authors:** Olga A. Kolesnikova, Dmitry A. Shikvin, Arina O. Antonova, Anna M. Iureva, Elena N. Komedchikova, Anastasiia S. Obozina, Valeryia S. Kachan, Anna V. Svetlakova, Ilya D. Kukushkin, Victoria O. Shipunova

**Affiliations:** Moscow Center for Advanced Studies, Kulakova Str. 20, 123592 Moscow, Russia

**Keywords:** nanoparticles, non-invasive detection, invasive detection, in vivo imaging, magnetic resonance imaging (MRI), computed tomography (CT), inductively coupled plasma mass spectrometry (ICP-MS), photoacoustic imaging, surface-enhanced Raman scattering, clinical application

## Abstract

Background/Objectives: Nanoparticles have emerged as indispensable tools in modern biomedicine, enabling precise diagnostics, targeted therapy, and controlled drug delivery. Despite their rapid progress, the translation of nanoparticle-based systems critically depends on the ability to detect, quantify, and track them across complex biological environments. Over the past two decades, a wide spectrum of detection modalities has been developed, encompassing optical, magnetic, acoustic, nuclear, cytometric, and mass spectrometric principles. Yet, no comprehensive framework has been established to compare these methods in terms of sensitivity, spatial resolution, and clinical applicability. Methods: Here we show a systematic analysis of all broadly applicable nanoparticle detection strategies, outlining their mechanisms, advantages, and drawbacks, and providing illustrative examples of practical applications. Results: This comparison reveals that each modality occupies a distinct niche: optical methods offer high sensitivity but limited penetration depth; magnetic and acoustic modalities enable repeated non-invasive tracking; nuclear imaging ensures quantitative, whole-body visualization; and invasive biochemical or histological assays achieve ultimate detection limits at the cost of tissue integrity. These findings redefine how each technique contributes to nanoparticle biodistribution and mechanistic studies, clarifying which are best suited for translational and clinical use. Conclusions: Placed in a broader context, this review bridges fundamental nanotechnology with biomedical applications, outlining a unified methodological framework that will guide the rational design, validation, and clinical implementation of nanoparticle-based therapeutics and diagnostics. By synthesizing the field into a single comparative framework, it also provides an accessible entry point for newcomers in nanotechnology and related biomedical sciences.

## 1. Introduction

When Galileo first turned his telescope to the night sky in 1609, it was not the stars themselves that changed, but humanity’s ability to observe them. That act of observation transformed astronomy forever, laying the foundation for modern science [1]. Nanomedicine today finds itself at a comparable turning point. The nanoparticles (NP) already exist, and their potential to reshape diagnostics and therapy is undeniable. But just as astronomy needed new instruments to make sense of the cosmos, nanomedicine now depends on reliable tools to make the invisible visible—to detect, follow, and quantify nanoparticles within living systems. Without such methods, the most sophisticated nanomaterials risk remaining theoretical constructs, promising on paper yet unproven in practice. This central challenge connects two worlds: the domain of fundamental science, where nanoparticles are studied as models and experimental tools, and the clinic, where their translation depends on demonstrating safety, efficacy, and measurable benefit.

Nanotechnology has profoundly expanded the potential of modern medicine, opening new avenues for diagnosing and treating a wide spectrum of diseases [2,3]. Nanoparticles have already demonstrated their value in addressing socially significant conditions such as cancer [4], cardiovascular [5] and metabolic disorders [6], and neurodegenerative diseases [7]. In the clinical arena, several nanoparticle-based formulations are approved and routinely used, primarily as imaging contrast agents or nanocarriers for drug delivery [8,9]. At the same time, in fundamental research, nanoparticles serve as indispensable tools for probing biological processes, developing experimental therapies, and designing next-generation diagnostic platforms [10,11,12,13].

The ability to follow nanoparticle fate in living systems is central to both domains. In fundamental science, reproducible and sensitive detection methods are the cornerstone for studying nanoparticle biodistribution, clearance, and cellular interactions. Without reliable readouts, even the most sophisticated designs cannot be validated. In medicine, precise detection is equally critical—whether for monitoring therapeutic efficacy, guiding surgical decisions, or ensuring patient safety. Yet, despite a wide arsenal of existing technologies, no method is without limitations. Optical fluorescence imaging, for example, is highly sensitive but provides limited penetration depth due to tissue scattering, whereas nuclear techniques deliver exquisite sensitivity but expose the subject to ionizing radiation and rely on complex radiolabeling protocols.

Several reviews have addressed particular aspects of nanoparticle detection—for instance, optical imaging and image-guided therapy [14,15,16], radiolabeled nanomaterials for positron emission tomography (PET)/single photon emission computed tomography (SPECT) [17,18,19], magnetic particle tracking and quantification [20,21,22], especially magnetic resonance imaging-based techniques that rely on relaxation time changes induced by Fe, Gd, Co, Mn, Dy and other paramagnetic atoms, and many others. Broader overviews also exist, such as recent comprehensive analyses of nanoparticles for nanomedicine [23,24]. However, these reviews are typically limited either by focusing on a single imaging class or by omitting a systematic comparison across invasive and non-invasive approaches.

The central aim of this review is to bring together, in one place, the full spectrum of methods available for nanoparticle detection—spanning optical and acoustic approaches, magnetic and nuclear imaging, and mass spectrometric techniques. Rather than offering a simple catalog, the review explains the physical principles that underpin each method, outlines their detection limits and resolution, and, crucially, distinguishes between applications in fundamental research and those already moving into clinical practice.

By drawing on insights across physics, chemistry, biology, and medicine, the review is intended to be useful to two key audiences. For researchers in basic nanoscience, it shows which tools allow reliable in vitro and in vivo characterization of new materials. For those working closer to translation, it clarifies which methods have already entered routine clinical use, which remain confined to animal models, and what obstacles still need to be overcome. In this way, the review aims to act as a bridge between discovery and application, helping both communities navigate the rapidly evolving landscape of nanoparticle detection and better realize its potential for nanomedicine.

The first essential distinction in nanoparticle detection is between non-invasive and invasive approaches. Non-invasive methods track nanoparticles directly in intact organisms and are indispensable for following whole-body distribution, accumulation, and clearance over time. Non-invasive detection methods include optical methods (fluorescence, bioluminescence, photoacoustics) providing high sensitivity but limited depth and quantitative methods for whole-body imaging (magnetic resonance imaging (MRI), computed tomography (CT), PET, and SPECT). Together, these complementary approaches offer a comprehensive toolkit for multimodal tracking of nanoparticles in living organisms. Invasive techniques require sampling blood or organs, perturb the system, and preclude real-time monitoring. In particular, microscopy methods (confocal, light-sheet, atomic force, and electron microscopy) enable high-resolution visualization of NPs at the cellular and subcellular levels using fluorescent, plasmonic, or electron-dense labels. Cytometric and mass-spectrometry-based techniques, including flow cytometry, cytometry by time-of-flight (CyTOF), inductively coupled plasma mass spectrometry (ICP-MS), and laser ablation ICP-MS (LA-ICP-MS), provide single-cell and elemental quantification of NP uptake and tissue distribution. Local bio-chemical and histological assays, together with radiometric methods, allow correlation of NP accumulation with molecular and physiological responses. These approaches complement non-invasive imaging and provide detailed insight into nanoparticle–tissue interactions.

**Figure 1:**
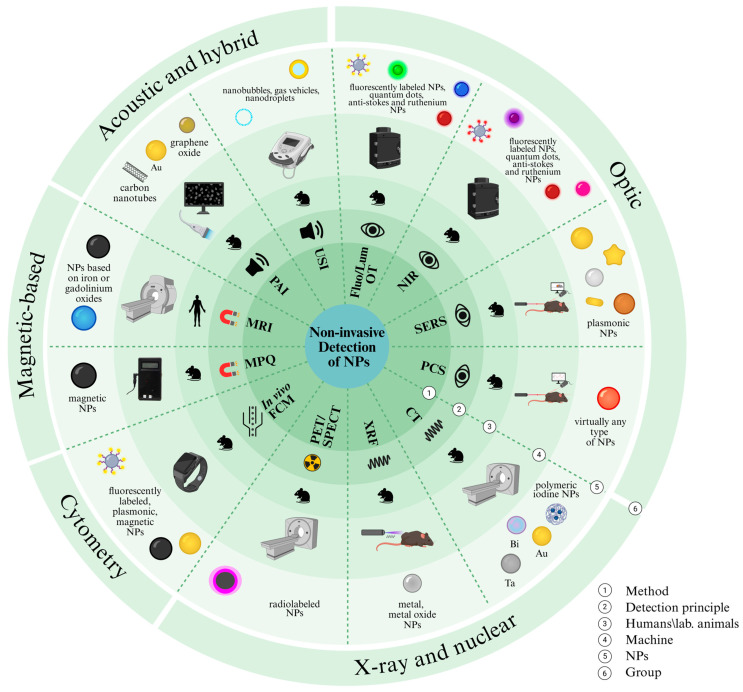
**A spectrum of non-invasive techniques for nanoparticles tracking in vivo.** Overview of major non-invasive modalities for in vivo nanoparticle detection and biodistribution analysis. The diagram integrates optical, magnetic, acoustic, X-ray, and nuclear techniques, highlighting their physical principles, typical nanoparticle labels, and instrumentation. Explanation of abbreviations: magnetic particle quantification (MPQ), magnetic resonance imaging (MRI), photoacoustic imaging (PAI), ultrasound imaging (USI), fluorescence/luminescence optical tomography (Fluo/Lum OT), near-infrared fluorescence imaging (NIR), surface-enhanced Raman scattering (SERS), photon correlation spectroscopy (PCS), computed tomography (CT), X-ray fluorescence (XRF), positron emission tomography (PET), single photon emission computed tomography (SPECT), in vivo flow cytometry (in vivo FCM). Created in BioRender. Shipunova, V. (2025) https://BioRender.com/5rk09bw.

**Figure 2:**
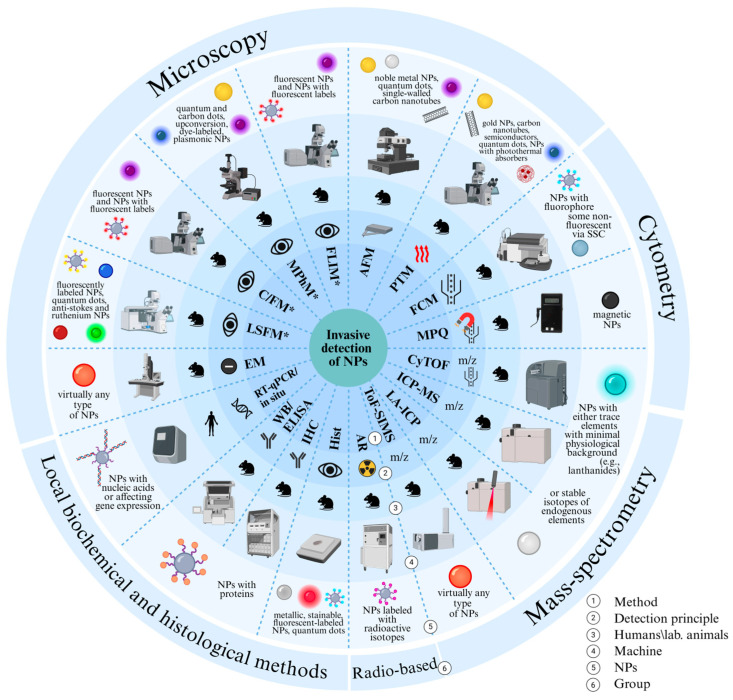
**A spectrum of invasive techniques for nanoparticles tracking.** The figure summarizes invasive and ex vivo analytical methods used for quantitative detection and localization of NPs in biological systems. Shown are microscopy, cytometry, mass spectrometry, biochemical, and radiobased approaches grouped by their physical principles. Explanation of abbreviations: electron microscopy (EM), light sheet fluorescence microscopy (LSFM), confocal/fluorescence microscopy (C/FM), multiphoton microscopy (MPhM), fluorescence lifetime imaging microscopy (FLIM), atomic force microscopy (AFM), photothermal microscopy (PTM), flow cytometry (FCM), magnetic particle quantification (MPQ), cytometry by time-of-flight (CyTOF), inductively coupled plasma mass spectrometry (ICP-MS), laser ablation inductively coupled plasma mass spectrometry (LA-ICP), time-of-flight secondary ion mass spectrometry (ToF-SIMS), autoradiography (AR), histology (Hist), immunohistochemistry (IHC), Western blot (WB), in situ hybridization (in situ). Methods applicable for in vivo detection are marked with *. Created in BioRender. Shipunova, V. (2025) https://BioRender.com/5rk09bw.

Figure 1 and Figure 2 outline the detection landscape, separating non-invasive and invasive strategies. In the sections that follow, each group is examined in turn with attention to physical principles, labeling requirements and compatible NP types, limits of detection, spatial/temporal resolution, quantifiability, throughput, biosafety, and translational status. This structure enables direct comparison across modalities and clarifies how ex vivo readouts validate signals obtained in vivo.

## 2. Non-Invasive Methods of Nanoparticle Detection In Vivo

Non-invasive modalities encompass magnetic, acoustic and hybrid, optical, and nuclear/X-ray imaging. Optical approaches (fluorescence, luminescence, near-infrared, and photoacoustic imaging) offer high sensitivity and temporal resolution but limited tissue penetration. Magnetic- and radio-based modalities (MRI, PET, SPECT, CT) enable whole-body imaging and quantitative biodistribution mapping, several of which are already approved for clinical use with contrast agents or NP-based formulations. Acoustic and hybrid techniques (ultrasound imaging, photoacoustic imaging) combine high spatial resolution with real-time monitoring capabilities. Experimental methods such as magnetic particle quantification (MPQ), surface-enhanced Raman scattering (SERS), and in vivo flow cytometry (FCM) are emerging in preclinical studies, offering unique advantages for label-specific detection at the cellular level. Together, these techniques form a complementary toolkit that bridges preclinical nanoparticle research and clinical translation, balancing sensitivity, safety, and depth of visualization.

**Figure 3:**
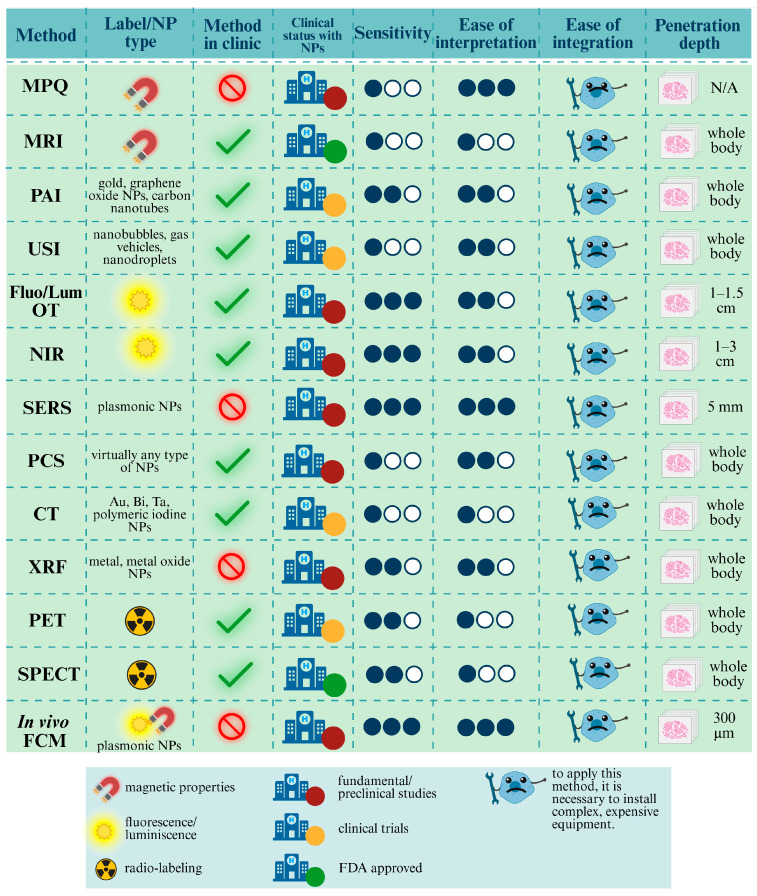
**Comparative advantages of non-invasive nanoparticles detection techniques in vivo in fundamental science and clinical application.** The table provides a comparative overview of major non-invasive imaging and analytical modalities used for NP detection and tracking in living organisms. Each method is evaluated by the type of NP label, clinical readiness, sensitivity, ease of data interpretation and lab integration, and penetration depth. Explanation of abbreviations: magnetic particle quantification (MPQ), magnetic resonance imaging (MRI), photoacoustic imaging (PAI), ultrasound imaging (USI), fluorescence/luminescence optical tomography (Fluo/Lum OT), near-infrared fluorescence imaging (NIR), surface-enhanced Raman scattering (SERS), photon correlation spectroscopy (PCS), computed tomography (CT), X-ray fluorescence (XRF), positron emission tomography (PET), single photon emission computed tomography (SPECT), in vivo flow cytometry (in vivo FCM). In the sections “ease of integration” and “ease of interpretation” we refer to the potential for the further development and translation of these modalities into clinical practice. Created in BioRender. Shipunova, V. (2025) https://BioRender.com/dfft74j.

Figure 1 provides a conceptual atlas of these techniques and their compatible labels; Figure 3 compares their sensitivity, interpretability, ease of integration, penetration depth, and clinical readiness.

In the subsections below we dissect each modality: from widely known MRI and CT to optical and photoacoustic methods, detailing detection mechanisms, practical detection limits in vivo, NP classes commonly used (e.g., iron-oxide, iodinated or high-Z metals, quantum dots, fluorophores, plasmonic absorbers), and typical use-cases (e.g., whole-body quantification versus high-sensitivity surgical guidance). We also highlight common pitfalls that affect interpretation (attenuation and scattering in tissue, photobleaching, label toxicity or instability, partial-volume effects) and outline best practices for multimodal study design.

### 2.1. Magnetic-Based Techniques

#### 2.1.1. Magnetic Resonance Imaging with Superparamagnetic Nanoparticles as Contrast Agents

MRI is a non-invasive imaging method based on the phenomenon of nuclear magnetic resonance. It allows acquisition of detailed images of internal anatomical structures with excellent soft tissue contrast. The first clinical MRI brain images were obtained in 1981 [25]. Today, MRI has become one of the most widely available imaging techniques, routinely used in medicine for disease diagnosis, treatment monitoring, and biomedical research [26,27].

The principle of MRI relies on the ability of nuclei with an odd number of protons, most notably hydrogen, to interact with an external magnetic field [28]. Hydrogen atoms are ideal probes because they are abundant in water and fat, the dominant components of the human body. When a patient is placed in the strong magnetic field of an MRI scanner, hydrogen protons align with this field, much like compass needles. Radiofrequency pulses are then applied to perturb these aligned protons, temporarily shifting them from equilibrium and causing them to absorb energy. Once the radiofrequency pulse ceases, the protons return to their original state, releasing energy in the form of an electromagnetic signal. This process, known as relaxation, occurs two main mechanisms: T1 (longitudinal) and T2 (transverse) relaxation. Because different tissues exhibit different relaxation times, MRI can generate strong contrast between them. The emitted signals are collected by specialized receiver coils and transformed into digital data, which are processed using Fourier transformation algorithms to reconstruct detailed three-dimensional anatomical images.

The core components of an MRI scanner include a high-field superconducting magnet, gradient coils that spatially encode signals, radiofrequency coils for transmission and reception, and a data processing unit. Clinical MRI systems typically operate with cryogenic superconducting magnets in the 0.5–3 T range, which is tens of thousands of times stronger than the Earth’s magnetic field [29]. Recently, 7 T instruments have entered clinical practice, offering improved signal-to-noise ratio, higher spatial and temporal resolution, quantitative parameter mapping capabilities, and shorter acquisition times [30]. With modern high-field scanners (≥3 T), spatial resolution can reach 0.05–0.5 mm in clinical settings [31] and down to 100 μm in animal studies [32]. The implementation of 7 T/9 T high-field MRI in the modern diagnostical centers has brought clinical diagnostics to a qualitatively new level over the past 20 years. Particularly, this method enables the visualization of microaneurysms, small perforating vessels, tumor texture and other sub-millimeter structures in the brain, providing neurosurgeons with the priceless information. A major advantage of MRI is the absence of ionizing radiation, making it safe for repeated in vivo use. However, without contrast agents, its sensitivity may be limited.

**Figure 4:**
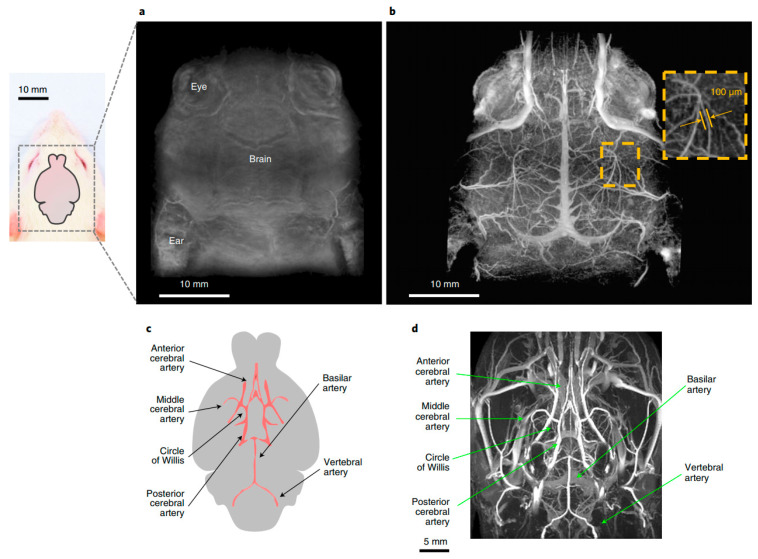
**High-resolution MRI of the rat head and brain with 100 μm spatial resolution.** (**a**) MRI scan before injection of SAIO, showing only gross anatomical structures. (**b**) MRI scan after SAIO injection, with visualization of brain vessels as thin as 100 μm. (**c**) Schematic of rat brain vasculature. (**d**) SAIO-assisted MRI of the rat brain, highlighting detailed vascular structures. Reproduced with permission from [32] © Springer Nature (2021).

Nanoparticles themselves can serve as powerful enhancers of MRI signals by directly modulating the relaxation properties of surrounding water protons. Unlike conventional small-molecule contrast agents, nanoscale formulations exploit their size, surface chemistry, and magnetic properties to produce stronger and more tunable effects. Paramagnetic gadolinium-based nanoparticles have been developed to improve T1-weighted imaging [33,34], whereas superparamagnetic iron oxide nanoparticles (SPION) form one of the most widely investigated classes for both T1 [35,36,37] and T2-weighted imaging [38,39,40], e.g., using SPIONs capped with poly(ethylene glycol)-derivatized phosphine oxide (PO-PEG) ligands as efficient T1 contrast agents for visualizing blood vessels down to 2 mm in diameter [41] or PEGylated 7 nm SPIONs as MRI contrast agent and hyperthermia induction for glioblastoma treatment [42,43]. Their exceptionally high magnetic moments allow them to locally distort the magnetic field and accelerate both T1 and T2 relaxation, generating strong signal amplification. Beyond the physics, nanoparticles can be engineered for selective accumulation in diseased tissues through surface coatings or biomolecular functionalization, enabling visualization not only of anatomy but also of specific pathological processes. This makes them particularly effective for detecting vascular networks, tumors, and sites of inflammation [44,45,46]. With optimized formulations, nanoparticle-assisted MRI can achieve detection sensitivity at millimolar concentrations, pushing the boundaries of what is measurable in vivo [47]. A representative example is shown in Figure 4, where supramolecular amorphous-like iron oxide nanoparticles (SAIO) enabled high-resolution MRI visualization of rat brain vasculature down to ~100 μm, demonstrating how nanoparticle-enhanced imaging can reveal fine vascular details that remain invisible without contrast supplementation [32]. Recent studies also demonstrate, that ultrasmall iron oxide nanoparticles with hydrophilic ligand exhibits high r1 and r2 relaxivity, comparable to clinical gadolinium-based contrast agents, making it suitable for both T1 and T2 contrast-enhanced MRI [48,49].

To improve stability and biocompatibility, SPIONs are often coated with polymers such as dextran or polyethylene glycol (PEG). Several formulations, including Ferumoxytol (Feraheme), Sinerem, and Resovist, have been approved by Food and Drugs Administration (FDA) for clinical imaging of vasculature, lymph nodes, liver, and tumors [50,51,52]. Beyond this, numerous studies have focused on functionalizing the surface of magnetic nanoparticles with antibodies or peptides to achieve selective accumulation in target tissues. This strategy enhances contrast specificity and reduces the required dose. For example, Ta et al. engineered antibody-functionalized SPIONs that selectively bind to activated platelets, enabling MRI detection of atherothrombosis [53].

Taking together, the use of nanoparticle-based contrast agents in MRI offers high T1- and T2-weighted image contrast and the possibility of tissue-specific imaging through surface functionalization. Compared with conventional gadolinium chelates, magnetic nanoparticles tend to show better biocompatibility, with reduced risks of nephrogenic systemic fibrosis and no brain accumulation, suggesting safer profiles for repeated administration [54]. Furthermore, recent advances in deep-learning–based image reconstruction and segmentation are significantly reducing the computational burden traditionally associated with MRI data processing. These approaches enable rapid generation and integration of high-resolution 3D MRI datasets, transforming workflows that previously required several hours into near-real-time procedures [55]. Challenges remain, however, including the optimization of dose and signal strength, since excessive particle concentration can cause signal darkening and complicate interpretation. Additionally, the need for stable coatings adds to manufacturing costs.

Overall, MRI has established itself as a highly effective method for the detection of nanoparticles in vivo. Superparamagnetic iron oxide nanoparticles remain the most widely studied and clinically applied probes, particularly for imaging of the vasculature, lymphatic system, liver, and tumors. In parallel, gadolinium-based nanosystems and emerging ultrasmall iron oxide formulations provide additional options for high-resolution T1- and T2-weighted imaging. Together, these platforms make MRI one of the most versatile and clinically relevant techniques for nanoparticle tracking and functional diagnostics.

#### 2.1.2. Magnetic Particle Quantification

MPQ is an original, highly sensitive method for magnetic nanoparticle detection, patented in Russia in 2000 [56]. MPQ is based on real-time quantitative measurement of the nonlinear magnetic response of magnetic nanoparticles in a liquid sample. The technique is compatible with various samples containing magnetic labels, including: cell suspensions for in vitro analysis using MPQ-cytometry [56,57], organs from sacrificed laboratory animals for ex vivo biodistribution studies [58], and real-time, non-invasive monitoring of nanoparticle circulation kinetics in vivo through tail vasculature of laboratory rodents [13]. The MPQ method operates by introducing a magnetically labeled sample into the inductive coil unit of the instrument, where it is exposed to a dual-frequency alternating magnetic field (f_1_ and f_2_). Then the system detects the sample’s nonlinear response at combinatorial frequencies f = n·f_1_ ± m·f_2_ (where n and m are integers, one of which may be zero) while varying the coil current. Moreover, the response amplitude is directly proportional to the concentration of nonlinear magnetic materials, such as superparamagnetic nanoparticles in the sample [59]. Meanwhile, magnetic signals from linear dia- and paramagnetic materials, commonly found in animal tissues (e.g., hemoglobin, blood, background signals from tissues), remain undetectable at these frequencies [60].

Thus, the MPQ method significantly reduces background signals from tissues and enables quantitative measurement of magnetic nanoparticles in the absence of additional labels (e.g., fluorescent, radionucleotide, etc.). For magnetic signal analysis, SPION are most commonly used [56,61,62]; however, the technique can also detect ferromagnetic Fe_19_Ni_81_ (permalloy) microdisks [63], metal–organic frameworks containing magnetic nanoparticles [64], polymer nanoparticles with magnetic labels [58], and liposomes with magnetic nanoparticles [65].

**Figure 5:**
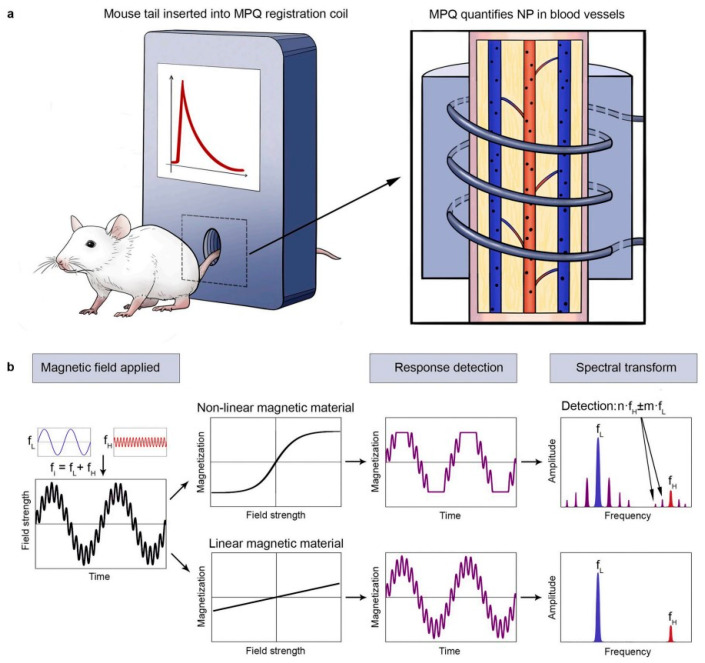
**MPQ registration of nanoparticle circulation kinetics.** (**a**) Experimental setup. Blood vessels are represented as veins (blue color) and arteries (red color). (**b**) Detection principle. The animal’s tail is placed inside a coil generating a magnetic field on frequencies f_1_ (f_L_, blue color on the graph) and f_2_ (f_H_, red color on the graph), magnetic nanoparticles are administered intravenously, their magnetic response is measured at a combinatorial frequency to detect particle concentration in tail veins and arteries. Reproduced with permission from [69] © Elsevier (2020).

The MPQ method demonstrates exceptional sensitivity comparable to quantification using γ-radioactive tracers such as the ^59^Fe isotope [66]. Due to its capacity for significant background signal suppression, the MPQ method achieves an in vitro detection limit of 60 zeptomoles [60] or 0.33 ng [56] of magnetic nanoparticles with an exceptionally wide linear dynamic range of 7 orders of magnitude. One of the key approaches for evaluating the in vivo behavior of nanoparticles is the analysis of their pharmacokinetics within the systemic circulation, which is schematically shown in Figure 5. Traditionally, such studies rely on invasive techniques involving serial blood sampling, which can distort the true clearance parameters due to induced reductions in blood volume and associated hemodynamic alterations. In contrast, MPQ enables the non-invasive, real-time monitoring of particle concentration dynamics in the bloodstream, facilitating its widespread application in fundamental scientific research. Specifically, for comparing the pharmacokinetic profiles of free nanoparticles versus erythrocyte-hitchhiking nanoparticles in vivo [13], studying prolongation of nanoparticles circulation in the bloodstream by macrophage blockade [59]. In a recent study, Gabashvili et al. successfully investigated, for the first time, the dynamics of murine macrophages in the bloodstream during their loading with magnetic nanoparticles in real-time [67]. Furthermore, MPQ technology enables the non-invasive investigation of nanoparticle biodegradation and quantitative assessment of their accumulation efficiency in the liver and spleen by positioning a detector coil over the murine abdominal region [68].

The original MPQ technique offers several distinct advantages, including compatibility with living organisms, high detection sensitivity for nanoparticles, high signal-to-noise ratio, the ability to utilize magnetic nanoparticles without additional labeling, and consequently simplified sample preparation. However, this method is inherently limited by its selective detection of nonlinear magnetic materials, which restricts its applicability to a narrow range of nanoparticle types. Furthermore, while the technique is effective for pharmacokinetic studies of nanoparticles, its application for non-invasive investigation of nanoparticle biodistribution in animal models remains significantly constrained.

### 2.2. Acoustic and Hybrid Techniques

#### 2.2.1. Ultrasound Imaging with Nanoparticle-Based Contrast Agents

Ultrasound imaging (USI) is a widely used diagnostic technique with low cost, high resolution, real-time monitoring modality, and portability. This method is based on the differences in backscattering of the applied sound waves by tissues and cavities. The use of contrast agents significantly widened the capabilities of the procedure in clinical diagnostics. Conventionally utilized FDA-approved contrast agents are microbubbles which suffer from insufficient extravascular penetration. Nanosized ultrasound contrast agents offer such advantages as: higher accumulation and longer retention in target tissue, for instance tumors, possibility for molecular imaging due to the targeting ligands on the surface, and theranostic applications [70].

**Figure 6:**
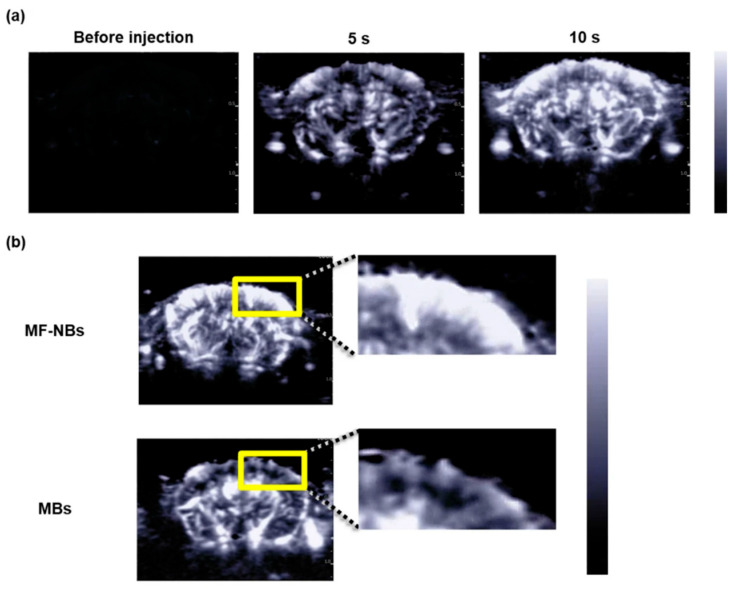
**Ultrasound imaging of brain microvessels using nanobubbles as a contrast agent.** (**a**) USI of mouse brain after i.v. injection of microfluidic nanobubbles (MF-NBs). (**b**) Comparison with microbubbles (MBs) as contrast agents for USI. The yellow square represents a scaled image. Reproduced from [74], CC BY.

Nanoagents for USI can be divided into three groups: nanobubbles, gas vehicles, and nanodroplets [71]. The first two contain gas inside, while nanodroplets are filled with liquid. One of the frequently used inner substance types are perfluorocarbons which belong to phase-change contrast agents [72]. The shell can be synthesized from various materials: lipid, liposomes, poly (lactic-co-glycolic) acid (PLGA), protein-based or surfactant-based nanoparticles. As an example of preclinical studies, Sabuncu et al. developed blinking nanoparticles for background-free USI [73]. Also, Yamaguchi et al. showed brain ultrasound visualization with nanobubbles with superior contrast compared to microbubbles (Figure 6) [74]. Despite the supposed benefits, the clinical translation currently faces many challenges [75]. Currently no nanobubble contrast agent is clinically approved for diagnostic imaging; only the one agent, RNS60, is under investigation as a therapeutic nanobubble formulation [76].

However, a novel technology comprising superparamagnetic iron oxide nanoparticles and ultrasound imaging—magnetomotive ultrasound (MMUS)—successfully reached Phase II trials [76]. The principle of MMUS is as follows: under the alternating magnetic field small nanoparticles move and cause local tissue displacement that is further visualized with ultrasound detector [77].

Ultrasound imaging with contrast agents is an emerging technique that enables deeper tissue visualization with improved sensitivity, although spatial resolution still decreases with depth. The disadvantages of the method include low quality of the image and low signal-to-noise ratio; moreover, qualified staff is required for obtaining reliable information. This stands in contrast to MRI, which achieves superior spatial resolution (0.05–0.5 mm), significantly exceeding the ~1 mm resolution limit of USI. Despite this drawback, USI’s real-time capabilities, portability, and cost-effectiveness secure its role as the gold standard for rapid pre- and post-operative assessment. Addressing USI’s fundamental limitation in penetration depth has therefore become a critical driver for innovation in contrast agents. Nanotechnology offers a viable strategy to achieve this by enabling the rational design of nanoparticles that can significantly boost USI signal strength and depth penetration. In the context of ultrasound, certain types of nanoparticles can be effectively detected, used as contrast agents or theranostic tools, but the approach needs to be optimized to meet the requirements of safety, cost-effectiveness and reproducibility.

#### 2.2.2. Photoacoustic Imaging

Photoacoustic imaging (PAI) is an emerging imaging modality that combines the penetration depth of ultrasound with the high spatial resolution of optical tomography. It is based on the photoacoustic effect—the generation of sound waves following light absorption by various endogenous or exogenous agents, such as hemoglobin. Absorbed light is converted into heat, leading to transient thermoelastic expansion and local pressure fluctuations that are detected as acoustic signals. PAI encompasses several approaches, including photoacoustic tomography (PAT) and photoacoustic microscopy (PAM).

**Figure 7:**
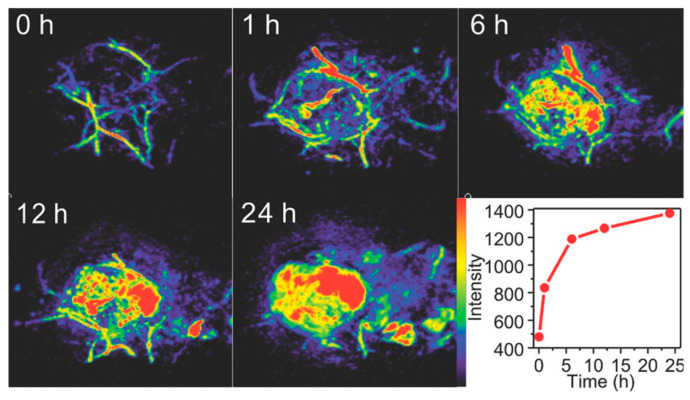
**Photoacoustic imaging of tumor vasculature in vivo in a murine model.** The indicated times correspond to images acquired before and after intravenous injection of Pd–Au nanoplates. Photoacoustic signals in tumor sites intensity changes during 24 h are presented in graph. Reproduced with permission from [80] © John Wiley and Sons (2014).

The use of nanoparticles with strong photothermal properties enables significantly enhanced image contrast and resolution [78]. Commonly investigated nanomaterials include gold nanostructures (spheres, stars, plates, rods), ruthenium oxide, iridium complexes, and other metal colloids [79]. For example, Chen et al. developed Pd–Au core–shell nanoplates coated with PEG, which exhibit strong near-infrared absorption and provide superior photoacoustic contrast enhancement [80]. As shown in Figure 7, prior to nanoplate injection only large vessels at the tumor site could be visualized with PAI, whereas post-injection a marked increase in photoacoustic signal intensity within the tumor was observed.

Overall, PAI holds considerable promise for accurate imaging of the vasculature across different organs. For instance, it has been investigated as a diagnostic alternative to X-ray mammography for breast carcinoma and may be preferable for some women due to reduced discomfort [81]. While its early application in oncology was largely motivated by the ability to detect tumor-induced angiogenesis, the development of targeted nanoparticle-based contrast agents has substantially broadened the potential applications of PAI in cancer diagnostics and beyond.

### 2.3. Optical Techniques

#### 2.3.1. Fluorescence Tomography for the Detection of Fluorescent Nanoparticles

Fluorescence tomography is a non-invasive bioimaging technique that enables three-dimensional visualization of the distribution of fluorescent markers in living tissues. In the context of nanoparticle detection, the method relies on reconstructing spatial maps of fluorescence signals generated when a biological object is illuminated with light of a defined wavelength [82].

During fluorescence tomography, the subject (e.g., a laboratory mouse) is illuminated with a laser or LED light source tuned to the absorption maximum of the fluorophore. Photons penetrate the tissue, where they undergo multiple scattering and absorption events. A fraction of these photons excites the fluorophore. According to Stokes’ law, the excited fluorophore emits light at a longer wavelength. In addition, anti-Stokes probes exist that emit light at shorter wavelengths. This effect is utilized in upconversion nanoparticles [83]. Such nanoparticles contain sensitizer ions, typically ytterbium, that absorb low-energy near-infrared photons and transfer the energy to activator ions, such as erbium or thulium. These activators can accumulate energy from multiple infrared photons and then release a single higher-energy photon. The emitted fluorescence photons scatter within the tissue, and only a small portion reach the surface, where they are captured by highly sensitive cameras, usually complementary metal-oxide semiconductor (CMOS) or electron-multiplying charge-coupled devices (EMCCD) detectors. A positioning platform allows the subject or the sources and detectors to be rotated, acquiring data from multiple angles to reconstruct a tomographic image.

The major challenge in fluorescence tomography of nanoparticles lies in reconstructing the precise location and concentration of the fluorophore from weak and highly distorted surface signals [84,85]. Photon trajectories in tissue are stochastic because scattering and absorption strongly depend on the optical properties of each organ and tissue type. As a result, light propagates differently through muscle, fat, or bone, leading to heterogeneous signal attenuation. This variability directly affects the accuracy of depth estimation: the same nanoparticle concentration located at different anatomical sites can appear with markedly different apparent intensities. Consequently, fluorescence tomography cannot provide a reliable quantitative readout of biodistribution across organs, and calculated parameters such as percentage of injected dose (%ID) based solely on fluorescence are inherently misleading. The intensity detected at the surface does not correspond linearly to the true amount of nanoparticles present, since secondary scattering and re-emission processes further alter the signal. To mitigate these issues, advanced light-propagation models and computationally intensive inverse algorithms are employed [82], but even with these corrections, fluorescence tomography remains best suited for qualitative or semi-quantitative assessment rather than precise quantification of nanoparticle biodistribution. For this reason, the method is increasingly combined with high-resolution modalities such as computed tomography, MRI, or magnetic methods, which can compensate for its depth- and tissue-dependent limitations [86,87,88].

Fluorescence tomography is particularly suited for detecting nanoparticles carrying fluorophores with high extinction coefficients, strong emission, and narrow spectral profiles [89]. A wide range of fluorophores can be coupled to nanoparticles, including fluorescent proteins, organic dyes like cyanines or fluorescein derivatives, and quantum dots. Quantum dots, semiconductor nanocrystals with size-tunable emission, are especially attractive due to their stability and brightness. Examples of quantum dot detection by optical tomography are virtually countless, reflecting the popularity of these probes in preclinical research [90]. For instance, Kang et al. demonstrated the biodistribution of carbon quantum dots in zebrafish tadpoles, with accumulation observed in the head, yolk sac, and tail [91]. These carbon dots showed low toxicity and good biocompatibility, underscoring their promise as clinical imaging probes. Another application is the labeling of stem cells with quantum dots to track their distribution in vivo in mice [92].

**Figure 8:**
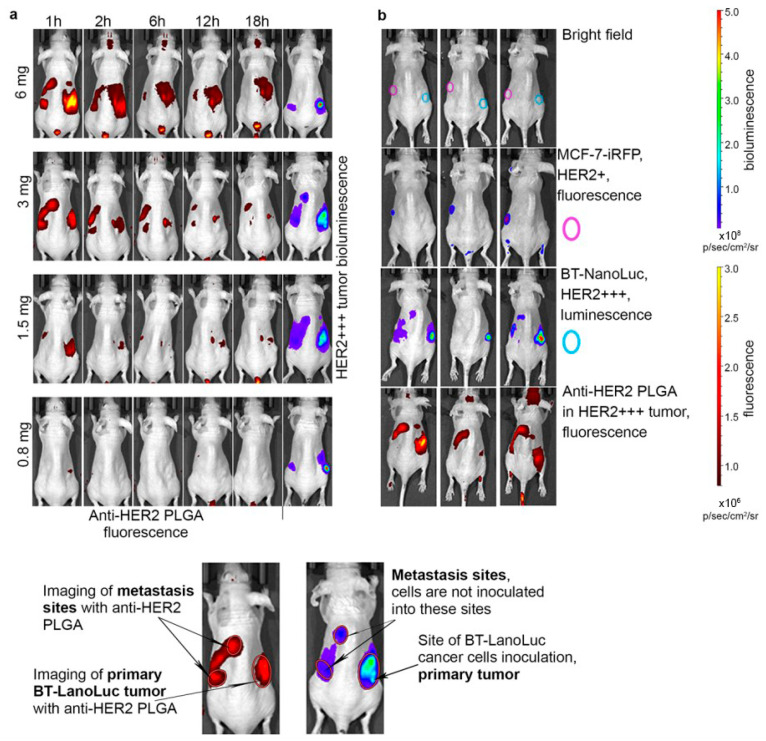
**Bioimaging properties of anti-HER2 PLGA nanoparticles in vivo.** (**a**) In vivo imaging of nanoparticles in mice with BT-NanoLuc tumors 1, 2, 6, 12, and 18 h post-injection. The primary BT-NanoLuc tumor and metastases are visualized by bioluminescence imaging. (**b**) Mice with two tumors: MCF7-iRFP tumors (left flank, pink circle, imaged via fluorescence detection) and BT-NanoLuc tumors (right flank, blue circle, imaged via bioluminescence detection) 1 h post-injection of nanoparticles. Reproduced with permission from [99] © American Chemical Society (2023).

Nanoparticles functionalized with covalently attached fluorophores provide an additional advantage: the ability to carry multiple dye molecules per particle, thereby amplifying the signal compared with single dye molecules. Numerous examples of such systems have been reported, including the detection of polymeric [92,93], magnetic [44], silver [94], silica-based, and many other nanoparticle platforms, highlighting the versatility of this strategy in optical fluorescence tomography [95]. Fluorescein-based derivatives are most often used in optical fluorescence tomography, either covalently bound to nanoparticles or encapsulated within them. Silicon nanoparticles doped with fluorescent dyes are one example [96]. These particles provide intense and stable signals and can be surface-functionalized to target specific tissues. Similarly, nanoparticles doped with ruthenium complexes exhibit prolonged excited-state lifetimes, making them easier to detect against the background of tissue autofluorescence [97]. Preclinical animal studies have established fluorescence tomography as a powerful tool for nanoparticle tracking. It is widely applied in oncology research for tumor visualization and for monitoring therapeutic responses [98]. A representative example is shown in Figure 8, where targeted to human epidermal growth factor receptor 2 (HER2) fluorescent PLGA nanoparticles distributed in mice demonstrate selective tumor accumulation, highlighting the ability of fluorescence tomography to resolve nanoparticle biodistribution in vivo [99].

In clinical practice, the use of fluorescence tomography remains limited by the shallow penetration depth of emitted light, which restricts it to small animal imaging and superficial tissues in humans. Nevertheless, the sensitivity of this approach is extraordinary: modern systems can detect fluorophores at concentrations in the picomolar range (10^−12^ mol/L) [100]. To address the challenge of poor depth penetration and limited spatial resolution, several technological strategies have been proposed. One direction is the development of hybrid imaging systems that combine fluorescence tomography with high-resolution anatomical modalities such as computed tomography or MRI [87,101,102]. Another approach focuses on optimizing the excitation source: commercial platforms such as IVIS Spectrum CT (PerkinElmer, Shelton, CT, USA) and MS FX Pro (Bruker, Billerica, MA, USA) employ xenon lamps, but their performance is limited because xenon emission falls sharply above 700 nm, making them less suitable for deep-tissue imaging. By contrast, laser- or LED-based excitation systems, for example LumoTrace FLUO (Abisense, Sochi, Russia), provide stable high-intensity illumination in the near-infrared region and thus improve penetration depth and imaging quality [103].

Taken together, these technological advances expand the potential applications of fluorescence tomography, yet the method as a whole retains inherent strengths and weaknesses that must be considered when applied to nanoparticle detection. The advantages of fluorescence tomography include its extremely high sensitivity, lack of ionizing radiation, relatively low equipment cost compared to positron emission tomography (PET) or MRI, and the possibility of long-term, real-time studies in the same animal. Its main drawbacks stem from the physics of photon scattering in tissue: limited resolution (typically 1–2 mm, rapidly degrading with depth), shallow penetration (generally ≤1–1.5 cm in small animals), and the need for fluorescent agents approved for medical use. In summary, fluorescence tomography provides a highly sensitive and technically accessible method for in vivo detection of nanoparticles, although its performance is ultimately constrained by photon scattering and tissue penetration depth.

#### 2.3.2. Near-Infrared Fluorescence Imaging

In the previous section, optical fluorescence imaging was introduced in the context of nanoparticle detection in vivo. Within this broad class of optical methods, a distinct variant deserves special emphasis: near-infrared (NIR) fluorescence imaging. By shifting excitation and emission into longer wavelengths, this approach partially mitigates the limitations of visible-range fluorescence, providing better tissue penetration and lower background autofluorescence.

For clarity, the bioimaging field divides the spectrum into NIR-I (650–950 nm) and NIR-II (1000–1700 nm), the latter also known as the short-wave infrared (SWIR) window. Within NIR-II, sub-ranges such as NIR-IIa (1000–1300 nm) and NIR-IIb (1500–1700 nm) are often distinguished. While NIR-I dominates clinical use today, NIR-II/SWIR has gained growing attention because reduced scattering and absorption in this range allow, in principle, deeper tissue visualization. Still, photon scattering imposes a fundamental limit: even in these “optical windows,” penetration rarely exceeds a few centimeters [104].

Instrumentation is similar to fluorescence tomography but employs laser or LED sources tuned to NIR wavelengths and filters matched to the emission spectrum of the fluorophore. A dedicated class of probes has been developed [105]. The most widely used are cyanine dyes such as indocyanine green (ICG), Cy5.5, Cy7, methylene blue, and IRDye800CW [106]. Only ICG and methylene blue are FDA-approved. To boost signal intensity, nanoparticles, e.g., liposomal [107], polymeric [108], magnetic and other inorganic structures [109] are frequently labeled with these dyes. Quantum dots based on PbS, PbSe, Ag, or In have also been explored [110], though their potential toxicity raises translational concerns.

**Figure 9:**
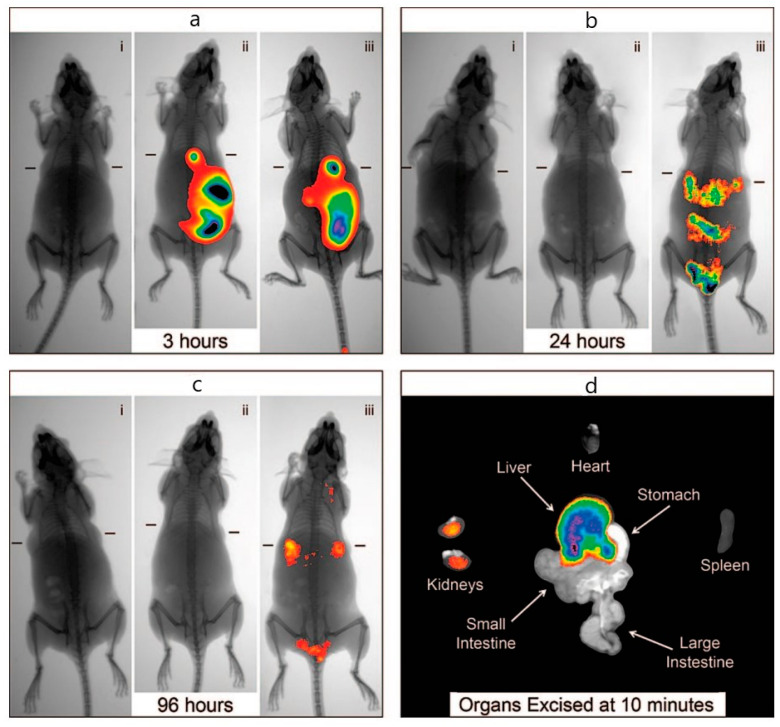
**NIR transillumination images of mice bearing subcutaneous breast tumors after injection of indocyanine green-loaded calcium phosphate nanoparticles (ICG-CPNPs).** (**a**) Fluorescence signals at 3 h compared with controls: (i) blank **calcium phosphate nanoparticles** (CPNPs), (ii) free **indocyanine green** (ICG), and (iii) PEGylated ICG-CPNPs. (**b**) Free ICG shows no detectable signal at 24 h (ii), whereas PEGylated ICG-CPNPs (iii) retain strong emission even at 96 h. (**c**) Tumor-localized fluorescence is evident 24 h post-injection with PEGylated ICG-CPNPs. (**d**) Ex vivo organ imaging 10 min after injection illustrates predominant biliary clearance with minimal renal involvement. Reproduced with permission from [122] © American Chemical Society (2008).

In preclinical models, NIR imaging has become a standard for in vivo monitoring [111]. Applications include tracking the tumor accumulation of gold nanoparticle clusters [112] and following immune cell migration: cytotoxic T-lymphocytes labeled with Cy5.5 can be reinfused and monitored as they home to lymph nodes, inflammatory sites, or tumors [113]. Other uses include angiography and perfusion studies in small animals [114]. More recently, probes emitting in the NIR-II/SWIR window have shown promise for deeper tissue visualization [115]. A representative illustration of NIR tomography is provided in Figure 9.

Clinical translation of NIR tomography is ongoing but selective. Early reports demonstrated feasibility for breast cancer imaging [116]. The most established clinical application is sentinel lymph node mapping in breast cancer and melanoma [117,118,119], where ICG drainage is visualized intraoperatively with NIR cameras integrated into surgical microscopes or handheld systems. Another emerging use is delineation of tumor margins during resection [120].

Resolution and sensitivity remain tightly coupled to tissue depth and probe design [121]. For superficial structures (≤1–3 cm), NIR-II probes can achieve ~50 µm resolution, but at greater depths scattering rapidly reduces it to ~0.5 mm. Sensitivity is comparable to fluorescence tomography, with detection limits in the picomolar range (10^−12^ M), equivalent to only a few thousand labeled cells or nanoparticles [100].

The main advantages of NIR imaging are its safety (non-ionizing radiation permits repeated measurements), relatively low cost compared to PET or MRI, equipment portability, and suitability for real-time intraoperative use. However, limitations include strong depth dependence of signal, the impossibility of tomographic reconstruction, and the very limited set of clinically approved dyes. Thus, NIR imaging should be considered a complementary rather than competing modality alongside CT or MRI.

#### 2.3.3. Light Sheet Fluorescence Microscopy

Light sheet fluorescence microscopy (LSFM) is a high-speed optical imaging method in which a specimen is illuminated by a thin sheet of light oriented orthogonally to the detection axis [123]. Unlike confocal or two-photon microscopy, which scan a focused point across the sample, LSFM excites only a thin plane (typically 1–10 μm) and records fluorescence with a sensitive camera (sCMOS or EMCCD) placed at 90°. This geometry reduces photobleaching and phototoxicity, making LSFM suitable for long-term in vivo experiments. Core components include a laser or LED excitation source, optics to generate the light sheet, the sample embedded in an immersion medium (e.g., hydrogel), a high-NA detection objective, a sensitive camera, and a sample positioning system for multi-angle acquisition [124].

For nanoparticle detection, LSFM relies on fluorescent labeling. Nanoparticles must either be intrinsically fluorescent (e.g., quantum dots) or conjugated with dyes such as Cy5, Alexa Fluor, or fluorescent proteins [125]. Labels can be incorporated into the particle matrix or covalently attached to the surface.

**Figure 10:**
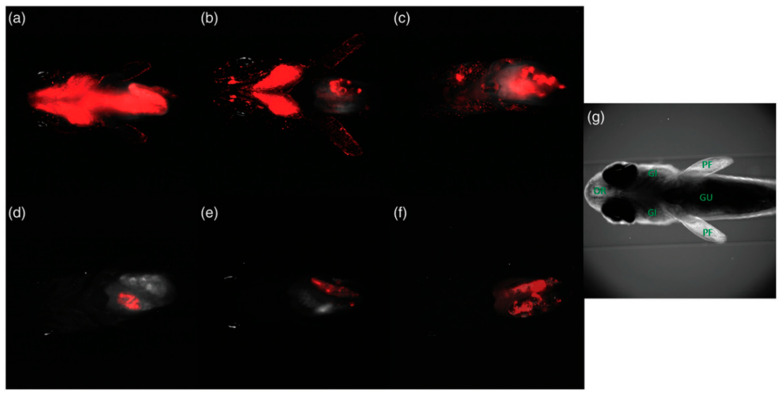
**Light sheet microscopy images of zebrafish exposed to fluorescent polystyrene nanoparticles** for 1, 3 and 7 days through the aqueous phase (**a**–**c**), and pre-exposed brine shrimp (**d**–**f**) as diet, respectively. The red signal corresponds to fluorescent polystyrene nanoparticles showing nanoparticles accumulation in different organs and tissues. (**g**) A transmission image of a control fish for orientation. OR: olfactory region; GI: gills; PF: pectoral fin and GU: gut. Reproduced with permission from [126] © Taylor & Francis (2017).

**Figure 11:**
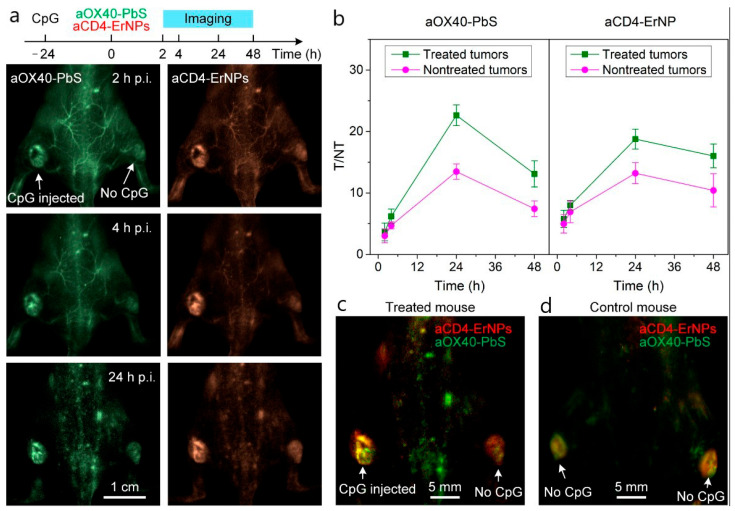
**Structured illumination light-sheet fluorescent microscopy images showing migration of CD4+ T cells labeled by functionalized nanoparticles to a CpG-injected tumor**. ((**a**), top) Treatment and imaging schedule. BALB/c mice were implanted subcutaneously with CT26 tumor cells on both the right and left hindlimbs. Cytosine-phosphate-guanine (CpG) was injected into one tumor site 24 h before intravenous injection of aOX40-PbS and aCD4-ErNPs. ((**a**), bottom) Wide-field NIR-IIb fluorescence images of a mouse with left tumor treated with CpG at various timepoints after injection of aOX40-PbS and aCD4-ErNPs. (**b**) Tumor-to-normal-tissue (T/NT) ratios of aOX40-PbS and aCD4-ErNPs from 2 to 48 h after injection. (**c**,**d**) Two-plex molecular imaging of mice at 24 h injection of aCD4-ErNPs (red) and aOX40-PbS (green). (**c**) Image of a mouse with left tumor injected with CpG. (**d**) Image of a control mouse without CpG treatment. Reproduced with permission from [128] © National Academy of Sciences (2021).

The method is widely applied to track biodistribution, circulation, and clearance of nanoparticles in vivo over hours to days. Transparent model organisms such as zebrafish [126] and *C. elegans* [127] are especially suited for real-time imaging. An illustrative example of fluorescent polystyrene nanoparticles biodistribution in zebrafish is shown in Figure 10. Furthermore, integration of structured illumination using digitally scanned laser beams to LSFM provides higher spatial resolution and allows to detect nanoparticles in non-transparent animals such as mice [128]. A representative example is shown in Figure 11, where anti-T cell functionalized erbium nanoparticles and PbS quantum dots are detected in the lymphatic system and particularly in tumors of mice bearing murine colorectal carcinoma after injection of a TLR-9 agonist. LSFM is also used for ex vivo imaging of glycerol-cleared organs [129] and for in vitro tumor cell assays with nanoparticle binding [130]. The technique achieves high detection sensitivity due to reduced out-of-plane background, though in vivo performance is limited by light scattering and tissue autofluorescence [123]. Typical spatial resolution is ~300–400 nm laterally and ~1 μm axially [131]. Frame rates can exceed 1000 fps, enabling visualization of nanoparticle dynamics in blood flow [131].

The main strengths of LSFM are fast volumetric imaging, low photodamage, and the ability to generate real-time 3D datasets. Its limitations are equally important: samples must be transparent or optically cleared, preparation is often complex, and fluorescent labeling of nanoparticles can alter their behavior in vivo. Finally, LSFM systems remain expensive commercial platforms requiring significant technical resources.

#### 2.3.4. Surface-Enhanced Raman Scattering Tomography for Nanoparticle Detection

SERS is a spectroscopic method that enhances the weak Raman scattering signals of molecules adsorbed on or located near metallic surfaces. When light interacts with matter, the vast majority of photons undergo elastic scattering, known as Rayleigh scattering. Only about 1 in 10 million photons undergoes inelastic scattering, producing what is known as the Raman effect. Because the intrinsic Raman signal is very weak, it has historically limited the broader application of this technique. Signal enhancement by SERS overcomes this limitation, dramatically improving detection efficiency and expanding the range of applications [132].

The precise mechanism of SERS enhancement remains a subject of scientific discussion. Two main theories are typically distinguished: the electromagnetic and the chemical enhancement mechanisms. It is now generally accepted that electromagnetic enhancement, arising from the excitation of localized surface plasmons on nanostructured metallic surfaces, is the dominant factor in most SERS processes [133].

Raman tomography is a hybrid technique that combines Raman spectroscopy with diffuse optical tomography, enabling reconstruction of the spatial distribution of chemical species within an object, including in vivo applications using nanoparticles. SERS nanoparticles typically comprise a metallic core responsible for localized surface plasmon resonance, a Raman reporter molecule providing a distinct Raman signal, and a stabilizing shell that improves nanoparticle stability and biocompatibility. Frequently, a targeting moiety is also incorporated to ensure selective accumulation in specific tissues [134]. This strategy has enabled non-invasive diagnosis of malignant tumor [135].

The main components of a Raman tomograph include: a laser source (usually tuned to one of the biological transparency windows [136], an optical scanning system, fiber optics, filters, and a spectrometer. For SERS tomography, nanoparticles such as gold [80,137], silver [138], or copper-based systems [139] are often used.

Morphological engineering of plasmonic nanoparticles, tuning them to specific localized surface plasmon resonance (LSPR) frequencies, further broadens their applications by improving sensitivity and spatial resolution. Anisotropic or branched morphologies such as nanorods [140], nanostars [141], and nanoshells [142] have expanded the use of the near-infrared optical windows.

**Figure 12:**
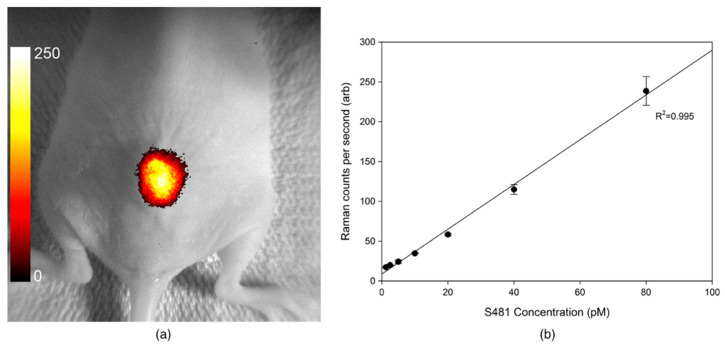
**In vivo detection limits of SERS nanoparticles.** (**a**) Wide-field SERS image of 80 pM reporter molecules injected subcutaneously into the dorsal region of a mouse, overlaid with a white-light optical photograph. (**b**) Serial dilutions of S481 in vivo (three replicates) demonstrated a detection limit of <2.5 pM with an exposure time of 5 s. Reproduced from [145], CC BY 3.0.

For in vivo applications, the ultra-high sensitivity of this method is particularly important. Nanoparticles can amplify Raman signals by 14–15 orders of magnitude [135], achieving detection at the nanometer scale (down to ~10 nm) [143]. For example, Zongyu Wu and colleagues demonstrated that ultra-bright SERS nanoparticles enabled detection through up to 4–5 mm of lung tissue [144]. Similarly, P. McVeigh and colleagues showed in in vivo mouse experiments that with a fixed scan time of only 5 s, the method achieved a limit of detection (LOD) below 2.5 pM [145], which is demonstrated in Figure 12.

Despite its clear advantages for in vivo imaging, the method has several limitations. Although non-invasive, SERS tomography is restricted by the penetration depth of near- and short-wave infrared light, which typically does not exceed ~5 mm [144]. Additional challenges are associated with the nanoparticles themselves. For instance, a substantial fraction of injected nanoparticles tends to accumulate in the spleen and liver, reducing delivery efficiency to the intended target site [146]. Moreover, certain types of nanoparticles, such as silver and copper nanostructures, can exhibit toxicity [147,148].

#### 2.3.5. Photon Correlation Spectroscopy and Related Methods

Photon correlation spectroscopy (PCS), also known as dynamic light scattering, is a noninvasive technique used to determine the size distribution of nanoparticles (typically 1 nm to ~1–2 µm) in colloidal suspensions. The method relies on fluctuations in scattered light intensity caused by Brownian motion of particles [149]. The decay time of the autocorrelation function is directly related to the diffusion coefficient, which in turn defines the hydrodynamic diameter via the Stokes-Einstein relation [149]. Larger particles produce slower fluctuations, smaller ones faster.

A typical PCS setup includes a coherent laser (commonly 632.8 or 532 nm), a quartz cuvette, collection optics at 90° or backscatter (173°), an avalanche photodiode or photomultiplier, and software that computes the autocorrelation function [150]. PCS remains the standard for nanoparticle sizing in liquids [151,152]. However, the method requires dilute, optically clear samples. Contaminants, aggregates, or strong scattering from cells and tissues obscure the nanoparticle signal [153], limiting its use in biological media to cleaned or simplified systems [154].

**Figure 13:**
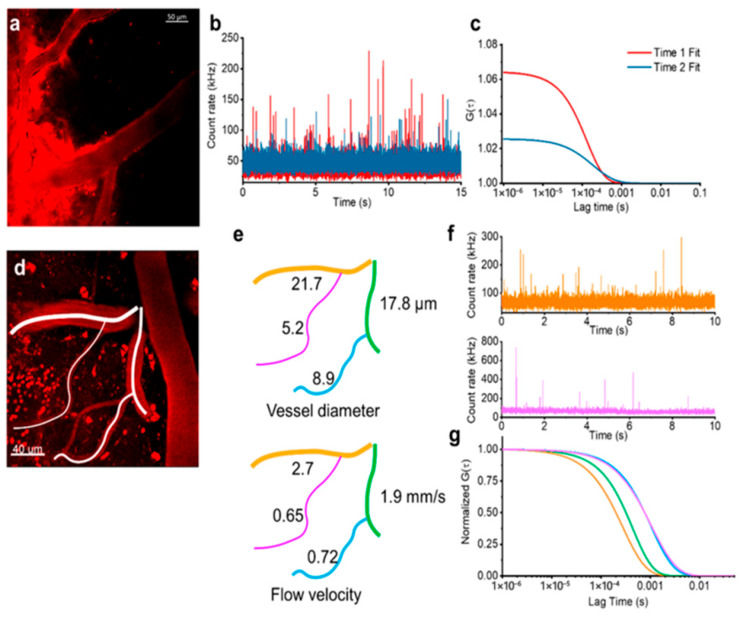
**In vivo fluorescence correlation spectroscopy of polymer nanoparticles in mouse brain vasculature**. (**a**) Fluorescent nanoparticle leakage from vessels after 1 h. (**b**) Fluorescence intensity traces directly after injection (red) and 1 h later (blue). (**c**) Corresponding autocorrelation functions (ACFs) showing concentration changes. (**d**) Two-photon image of labeled cerebral vasculature. (**e**) Vessel diameter and flow velocity relationship. (**f**) Intensity traces from a large vessel (21.7 µm, orange) and a capillary (5.2 µm, pink). (**g**) Corresponding ACFs illustrating flow velocity differences depending on vessel diameter (color matched to part **e**). Represented with permission from [164] © American Chemical Society (2020).

To extend applicability, two modifications are used. X-ray photon correlation spectroscopy (XPCS) replaces visible light with coherent synchrotron X-rays [155]. Shorter wavelength and higher penetration allow analysis of concentrated suspensions and ex vivo biological fluids, including nanoparticle aggregation, sedimentation, and protein corona formation [156,157,158,159]. The drawback is clear: access is limited to synchrotron facilities, and experiments are technically demanding. Fluorescence correlation spectroscopy (FCS) analyzes temporal fluctuations of fluorescence rather than scattered light [160]. A confocal volume of ~1 fL is illuminated; each labeled particle crossing it produces a burst of photons. Autocorrelation of these bursts yields diffusion coefficients, concentrations, and sizes. FCS can probe nanoparticles in vitro [161], ex vivo [162,163], and in vivo [164,165]. An illustrative example of in vivo nanoparticle detection and measurement of its concentration and flow rate in mouse brain vessels is shown in Figure 13. It has even been applied clinically, e.g., to detect amyloid-β aggregates in cerebrospinal fluid of Alzheimer’s patients [166]. The method reaches picomolar sensitivity but requires fluorescent labeling and is highly susceptible to artifacts such as photobleaching and triplet-state dynamics [167]. To summarize, PCS is fast, label-free, and ideal for routine quality control of monodisperse colloids, but unsuitable for complex biological samples. XPCS removes transparency constraints and enables studies of concentrated systems, but synchrotron dependence limits practicality. FCS provides unparalleled sensitivity and selectivity in biological contexts, but only with fluorescent probes and careful control of photophysics.

### 2.4. X-Ray and Nuclear Techniques

#### 2.4.1. Computed Tomography for the Detection of High-Z Nanoparticles

CT is a medical imaging technique based on the use of X-rays (highly collimated streams of high-energy photons produced when accelerated electrons strike a metal target) to acquire cross-sectional images of internal structures, which are then reconstructed into a three-dimensional model. CT provides detailed anatomical information and enables the detection of pathological changes such as tumors, hemorrhages, fractures, and inflammatory processes with high accuracy [168].

The technique relies on measuring the attenuation of X-ray beams as they pass through tissues of varying density [169]. The key components of a CT scanner are the X-ray tube and the detector array. The tube generates X-ray beam, which traverse the patient’s body, while detectors capture the attenuated radiation and convert it into electrical signals. Both the tube and detectors are mounted on a rotating gantry, which encircles the patient, while the patient table moves through the gantry to enable scanning of multiple slices. The acquired data are processed by reconstruction algorithms, commonly filtered back projection or iterative reconstruction methods, to generate tomographic cross-sections of the scanned region [170]. The denser the tissue, the higher the X-ray absorption, which appears as brighter regions on the CT image.

CT is characterized by rapid scan speed, the ability to perform three-dimensional reconstructions, and excellent visualization of bone and other high-density tissues. Clinical CT scanners typically achieve a spatial resolution of ~0.5–1 mm, whereas micro-CT systems designed for preclinical studies can reach resolutions down to 1–100 μm [168]. The main limitation of CT is its reliance on ionizing radiation, which carries potential risks for patients in the case of repeated or long-term imaging. In addition, CT has limited soft tissue contrast when densities are similar. To improve image quality, contrast agents are frequently used. Conventional iodinated solutions are the most common, but their small molecular size leads to rapid renal clearance and short blood circulation times [171]. To compensate, high doses are often administered, which can result in allergic reactions or nephrotoxicity [172]. These drawbacks have motivated the development of nanoparticle-based alternatives.

**Figure 14:**
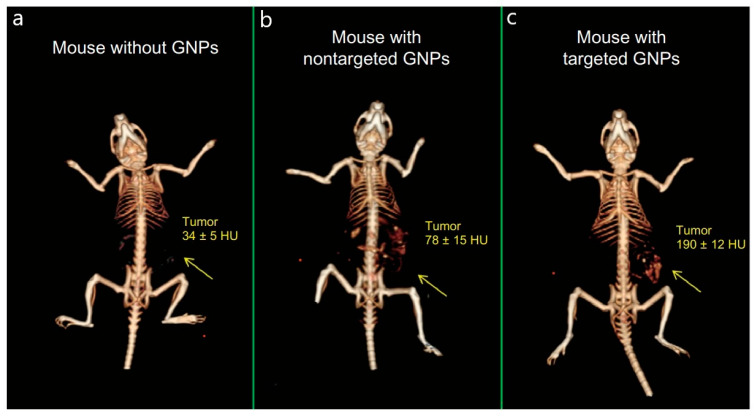
**In vivo CT detection of tumors using gold nanoparticles: comparison of passive and active targeting.** (**a**) CT image before nanoparticle injection. (**b**) CT image 6 h after injection of non-specific IgG-coated GNPs (passive targeting). (**c**) CT image 6 h after injection of anti-EGFR-coated GNPs (active targeting), showing clear tumor contrast enhancement. Reproduced from [180], CC BY-NC 4.0.

High-Z element nanoparticles are particularly well suited for CT because of their strong X-ray attenuation, making them ideal markers for in vivo detection [173,174]. Gold, bismuth, and tantalum nanoparticles, as well as liposomal or polymeric iodine-loaded particles, are actively investigated in preclinical studies to enhance CT contrast [175,176,177,178]. Compared with conventional iodinated solutions, these nanostructures exhibit higher X-ray attenuation coefficients, enabling sharper images at lower radiation doses. Moreover, nanoparticles display prolonged blood circulation and can be functionalized with targeting ligands, such as antibodies or peptides, to direct them to specific tissues, thereby increasing diagnostic accuracy [176]. For instance, in mouse experiments, PEGylated gold nanoparticles functionalized with anti-CD4 antibodies produced a twofold increase in CT contrast of lymph nodes compared with nonspecific antibody-coated particles, with the enhanced contrast persisting for up to 48 h [179]. An illustrative example is shown in Figure 14, where in vivo CT scans highlight the difference between passive and active nanoparticle targeting. While nonspecific IgG-coated gold nanoparticles provided only limited enhancement, anti-EGFR-coated formulations generated a clear and specific increase in tumor contrast [180].

Nevertheless, the application of nanoparticles as CT contrast agents is not without challenges. Potential toxicity of some nanomaterials, high production costs, scale-up difficulties, and the need for further safety validation remain important concerns. In summary, CT combined with high-Z nanoparticles provides a technically robust and highly sensitive approach for in vivo detection, enabling quantitative visualization of nanoparticle distribution with microscale spatial resolution.

#### 2.4.2. Radiolabeled Nanoparticles for PET, SPECT, and Cherenkov Luminescence Imaging

PET combined with either magnetic resonance imaging or computed tomography (PET/MRI or PET/CT) is an advanced diagnostic modality based on the high-resolution detection of a radiopharmaceutical tracer within the body. Integration with MRI enables precise localization of tumorous cells, damaged tissue, or inflammation in soft tissues and organs, whereas the use of CT allows for the visualization of pathological changes in bones and dense tissues, respectively [181].

A conventional PET study is based on the detection of positron emission from a specific radioactive label, particularly, ^18^F within fluorodeoxyglucose (FDG) [182]. Metabolically hyperactive cells, such as those found in malignant tumors and other pathologically changed tissues, exhibit markedly increased glucose uptake. Consequently, FDG, a glucose analogue, accumulates preferentially within these target tissues. Following administration, the radionuclide (e.g., ^18^F) undergoes β+ decay. The emitted positron travels a short distance in tissue before annihilating upon collision with an ambient electron. This annihilation event produces a pair of coincident, high-energy (511 keV) gamma photons propagating in opposite directions (180 degrees apart). A PET scanner is equipped with a circumferential array of detectors designed to simultaneously detect these paired annihilation photons. The detection of such coincidence events allows for the precise spatial localization of the radiotracer. Subsequent sophisticated computational algorithms reconstruct these signals into a three-dimensional tomographic map of the radiotracer’s distribution, which is then combined with the MRI or CT anatomical scan.

**Figure 15:**
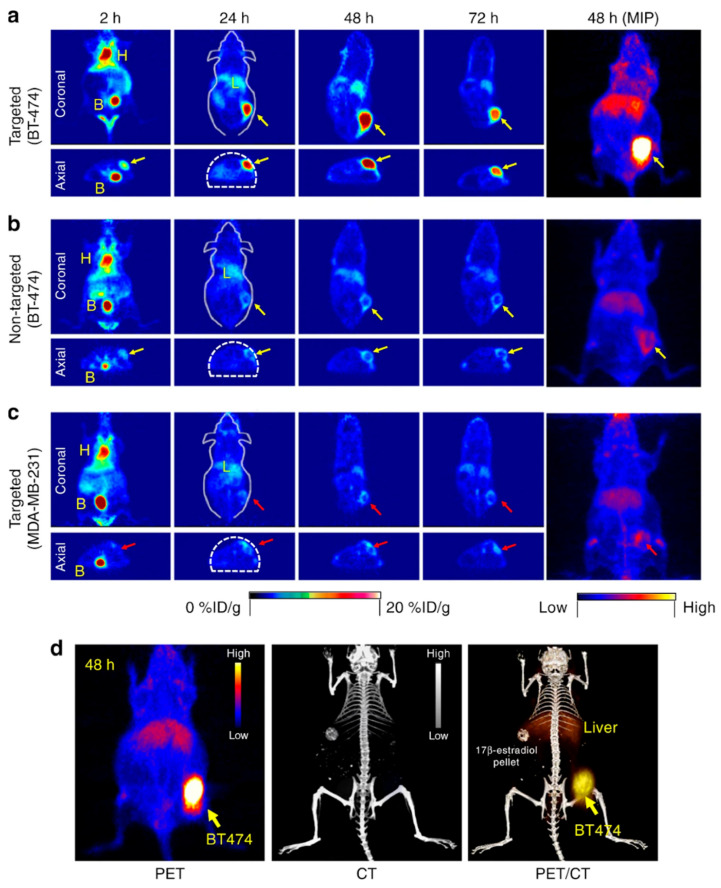
**In vivo high-resolution PET and PET/CT imaging of a HER2-positive murine model of breast tumor with targeted ultrasmall silica nanoparticles, radiolabeled with ^89^Zr**. (**a**) Targeted group in BT-474 mice. (**b**) Non-targeted group in BT-474 mice (**c**) Targeted group in MDA-MB-231 mice. (**d**) Representative MIP PET, CT, and PET/CT fusion images in a BT-474 tumor-bearing mouse. H—heart, B—bladder, L—liver, MIP—maximum intensity projection. The top line represents the time post i.v. injection. Reproduced from [195], CC BY 4.0.

The development of nanoparticle-based radiopharmaceutical tracers significantly expands the capabilities of PET, as the production and use of radioactive labels remain the main limiting factors at this stage in the development of the technique. The rational selection of the combination of the nanoparticle blood circulation half-life and the physical half-life of the radionuclide ensures high imaging efficacy while reducing the radiation dose [183]. Nanoradiopharmaceuticals can be functionalized with specific targeting molecules (e.g., peptides, antibodies) for the precision imaging of tumors, internal organs, and other structures [184]. For example, HER2-positive breast tumors were visualized in mice, using scFv-modified ultrasmall silica nanoparticles (Cornell dots, C-dots) (Figure 15). The incorporation of a radionuclide onto a nanoparticle can be achieved via direct labeling, indirect conjugation through a linker (bifunctional chelator), or encapsulation. Current chelation methods for radiotracers are sufficiently advanced to allow for reliable nanoparticle labeling, with binding efficiencies reaching up to 90% [185]. Furthermore, nanoparticles can be simultaneously modified with multiple agents for theranostic applications or multimodal diagnostics [186]. Conversely, radioactive labeling of nanoparticles is frequently employed in fundamental research to track the biodistribution of a nanomaterial in vivo using PET and to quantitatively assess its accumulation in the tumor [187].

As methods for attaching radioactive labels, including chemical linkers, are continuously being refined, a wide variety of nanoparticle types can be utilized as PET tracers [188]. To date, the only nanoradiopharmaceuticals in clinical trials are ultrasmall silica nanoparticles (Cornell dots, C-dots) labeled with ^64^Cu, ^89^Zr, or ^124^I [189,190]. These tracers can be used for diagnosing brain tumors or for pre-operative imaging of prostate tumors. The multifunctionality of these nanoparticles is noteworthy: the investigated C-dots are also modified with a fluorescent dye for optical imaging and a cRGDY peptide for targeting tumor cells [189]. Labels based on metal nanoparticles (especially gold) and metal oxides, including those that enhance MRI or CT contrast, are also being actively developed [191]. For PET imaging, liposomes, polymeric nanoparticles, carbon nanomaterials of various shapes, and quantum dots are being utilized [192].

The PET imaging itself possesses good spatial resolution—on the order of 2–4 mm in clinical scanners and below 1 mm in small-animal systems. Image quality depends on the characteristics of the radioactive label and the design of the detector sensors. Resolution can be further enhanced through fusion with MRI or CT (for instance, with attenuation correction) and with advanced computer processing [192]. The limit of detection is primarily determined by the efficiency of the nanoparticle’s radioactive labeling and can range from 0.1 to 10 nanomoles of the administered agent [193].

Another method for in vivo imaging and detection of nanoparticles based on a radioactive signal is SPECT. In contrast to PET, SPECT utilizes radioisotopes that emit a single gamma-ray photon upon decay, instead of a positron. Consequently, the primary isotopes used for this method are ^99^mTc, ^111^In, ^67^Ga, and ^177^Lu. Detection of the emitted photons is performed by collimators positioned opposite each other and rotating around the patient. SPECT also enables the reconstruction of a three-dimensional image of the body and can be used to track the biodistribution of radiolabeled nanoparticles.

Several nanoradiopharmaceuticals with the ^99^mTc isotope, namely albumin, sulfur, SnF_2_, and Re_2_S_7_ colloids, are already FDA-approved for clinical use [194]. As with PET, various types of nanoparticles labeled with a suitable isotope, such as PLGA, silica, and lipid nanoparticles, are utilized for SPECT [183]. The resolution of SPECT is slightly inferior to that of PET when using high-quality collimators (4–6 mm). Labeled nanoparticles can also be detected with high sensitivity—it is dependent on the object of visualization and radioactivity of the agent.

Cherenkov Luminescence Imaging (CLI) is an innovative optical method for imaging the biodistribution of a radioactive tracer in vivo, which is now beginning to be actively introduced into clinical practice [196]. This approach is based on the detection of visible or near-infrared light emission generated by charged particles (positrons or electrons) traveling faster than the phase velocity of light in a given medium (Cherenkov radiation, discovered by P. Cherenkov and S. Vavilov in 1934). Despite the same necessity of using a radioactive tracer, CLI offers advantages over PET and SPECT, including significantly lower equipment cost, real-time imaging capabilities, and rapid results [197]. CLI can detect radioisotopes undergoing not only β+ decay but also β− decay. Due to its low cost and rapid procedure time, CLI can be used as an adjunctive control method during radionuclide therapy without significantly increasing patient burden.

Cherenkov radiation was first detected in vivo by Robertson’s group in 2009 using the positron emitters ^89^Zr and ^18^F [198]. Since the majority of Cherenkov emission is in the blue region of the spectrum, the detection depth of the tracer within the body is limited to a few millimeters. The use of nanoparticles to enhance and shift the signal toward the red and even infrared regions significantly expands the potential applications of CLI for bioimaging [199]. One actively researched approach involves using a radiotracer in conjunction with quantum dots (e.g., self-illuminating ^64^Cu-doped CdSe/ZnS QDs [200], whereby the QDs are excited by the blue light of Cherenkov radiation and emit longer-wavelength light [201]. This phenomenon is termed Cherenkov Radiation Energy Transfer (CRET), by analogy with Förster resonance energy transfer (FRET). For CLI, radiolabeled gold nanoparticles, lanthanide-doped nanophosphors, and other materials are also used [202]. As mentioned above, the resolution of CLI and the limit of detection of nanoparticles are highly sensitive to the depth of the target region (e.g., a tumor) because biological tissues strongly absorb visible light. For targets close to the skin surface or when the emission is shifted to the infrared range, the spatial resolution can reach less than 1 mm [203].

Thus, radioactivity-based methods—PET, SPECT, and CLI—are highly sensitive, quantitative visualizing techniques for detecting nanoparticles with minimal or no background signal. The main disadvantage of these methods is the necessity of using a radioactive isotope, which may be unavailable in a laboratory due to financial or technical constraints, and which exposes the animal or patient to radiation, albeit at a low dose.

#### 2.4.3. X-Ray Fluorescence

X-ray fluorescence (XRF) is a method based on the excitation of sample atoms by X-rays. The source of X-ray radiation is typically an X-ray tube, although synchrotron radiation can also be used. When an incident X-ray photon ejects an electron from an inner shell, the resulting vacancy is filled by an electron from a higher orbital. This transition releases a photon with an energy characteristic of the specific element. This secondary (fluorescent) X-ray signal is registered by a detector, commonly consist of Germanium (Ge), lithium drifted silicon (SiLi) or silicon drift detector (SDD) [204]. The method can be applied for both qualitative and quantitative determination of nanoparticles in tissues, with measurements performed either in vivo or ex vivo.

Effective quantitative detection of nanoparticles using X-ray fluorescence was demonstrated for gold NP by K. Ricketts and colleagues [205]. Their work showed that XRF-based detection of gold NPs allows discrimination between tumor and non-tumor tissue based on concentration differences of up to fivefold. The experiments were performed both in vivo and in vitro. Similarly, Yu Kuan and co-workers demonstrated the multiplexing potential of XRF using gold nanoparticles [206]. In their study, XRF enabled quantitative mapping of gold, gadolinium, and barium. The detectable concentration of gold ranged from 0.06% to 0.1% Au in an Eppendorf tube for SDDs, while CdTe detectors failed to register such low concentrations. Further experiments showed that the detection limit for SDD was 0.007% Au, sufficient for detecting gold in small-animal studies.

Detection of even lower Au concentrations was reported by M. Ahmad and colleagues [207], who demonstrated that the detection threshold depends on the angle of the detector. The lowest detectable concentration (0.25%) was achieved when the detector was positioned at 60° (forward scattering) and 90° (side scattering).

**Figure 16:**
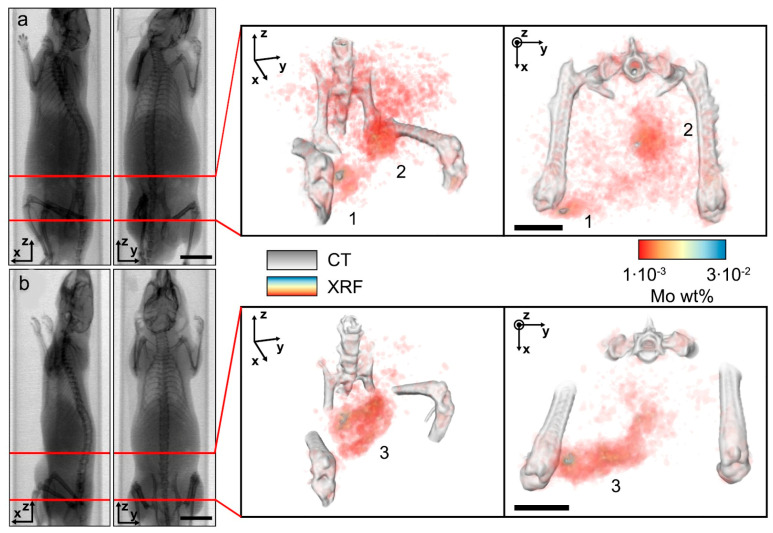
**X-ray fluorescence tomography.** Tumor detection using XRF tomography shown as overlays of three-dimensional XRF and CT images of tumors in two mice. The imaged regions correspond to the areas above the hind limbs, outlined by red lines on the left-side projections. Scale bar = 5 mm. (**a**) Tumor with a volume of 22 mm^3^ (1) and urinary bladder (2). (**b**) Tumor with a volume of 151 mm^3^ (3). Reproduced from [209], CC BY 3.0.

X-ray fluorescence has also proven highly effective for in vivo nanoparticle visualization. For instance, G. M. Saladino et al. demonstrated in vivo and in situ visualization in mice [208]. Detection was achieved at depths up to 2 cm, with particles visualized at concentrations of 50 μg/mL. The nanoparticles tested included MoO_2_, Rh, and Ru coated with a silica (SiO_2_) shell, with MoO_2_ nanoparticles showing the best performance for imaging. The reported spatial resolution was ~100 μm in live rodents, superior to X-ray or CT imaging. J. C. Larsson and colleagues also studied molybdenum-based nanoparticles in mice [209], evaluating biodistribution in tumor-free animals as well as visualization of neuroblastoma xenografts (SK-N-BE(2) cells). Images were obtained by overlaying CT scans with X-ray fluorescence tomography data (Figure 16). In these biodistribution experiments, most nanoparticles accumulated in the liver and lungs. Tumor accumulation data correlated well with measurements obtained by ICP-MS.

Previously, XRF was considered unlikely to be applicable in preclinical and clinical studies. However, significant technical advances now make this method highly promising for such applications [210]. The advantages highlighted earlier include high resolution (superior to CT), the possibility of in vivo use, and minimal sample preparation. Importantly, XRF allows for simultaneous tracking of multiple labels [211]. Nevertheless, the method has certain limitations: successful imaging requires nanoparticles with a high atomic number (Z > 23) [212], relatively high radiation doses [213], and expensive modern instrumentation.

### 2.5. Flow-Based and Cytometry-like Techniques

#### In Vivo Flow Cytometry

In vivo flow cytometry (IVFC), a technique that enables real-time detection of fluorescently labeled nanoparticles directly in the bloodstream without sample collection, has emerged recently. By focusing a laser on superficial blood vessels, IVFC records fluorescence signals from circulating particles, allowing dynamic monitoring of their distribution, clearance, and aggregation in living organisms. An IVFC-based device has already been proposed for use in patients with blood loss or for liver function studies [214].

Since the development of the first in vivo fluorescence cytometer by Lin’s group in 2004, this method has been widely applied in preclinical research [215,216]. For example, Wei et al. used in vivo flow cytometry to non-invasively monitor fluorescently labeled nanoparticles in mouse ear vessels, showing that PEG-5K-modified polylactic nanoparticles exhibited significantly longer circulation times and formed fewer and smaller aggregates in the bloodstream compared to PEG-3K-modified ones. While this approach offers high temporal resolution and eliminates the need for blood sampling, it is limited by the shallow detection depth of fluorescence IVFC (~300 µm), the inability to resolve aggregate morphology, and low sensitivity to aggregates smaller than 500 nm [217].

**Figure 17:**
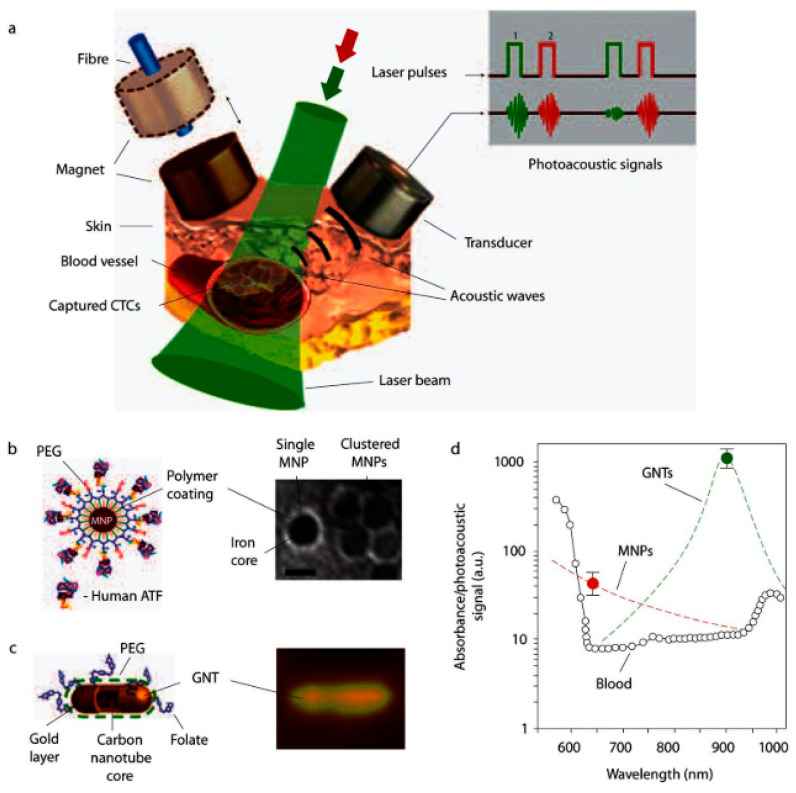
**In vivo magnetic enrichment and photoacoustic detection of circulating tumor cells.** (**a**) Scheme of CTC detection. (**b**) Magnetic nanoparticle (MNPs) design and transmission electron microscopy (TEM) image. (**c**) Gold–carbon nanotube (GNTs) design and atomic force microscopy topography image. (**d**) Photoacoustic spectra of ~70 µm veins in the mouse ear (open circles). Absorption spectra of MNPs and GNTs (dashed red and green curves) are normalized to photoacoustic signals from CTCs labeled with MNPs (filled red circle) and GNTs (filled green circle). Reproduced with permission from [219] © Springer Nature (2009).

In vivo flow cytometry can utilize not only fluorescence detectors but also detectors of scattered light, acoustic waves, Raman scattering, or heat emission [218]. These approaches enable label-free detection of nanoparticles on circulating cells. Particularly, Galanzha et al. introduced magnetic nanoparticles for capturing and detecting circulating tumor cells (CTCs) in vivo (Figure 17) [219].

In summary, IVFC employs different types of nanoagents, especially fluorescently labeled nanoparticles with diverse core materials and plasmonic nanoparticles with high photoacoustic contrast, depending on the detection modality. This non-invasive technique provides real-time information on the circulation of nanoparticles and their interaction with blood cells, offering both qualitative and quantitative data.

## 3. Ex Vivo and Invasive Methods for Nanoparticle Detection

**Figure 18:**
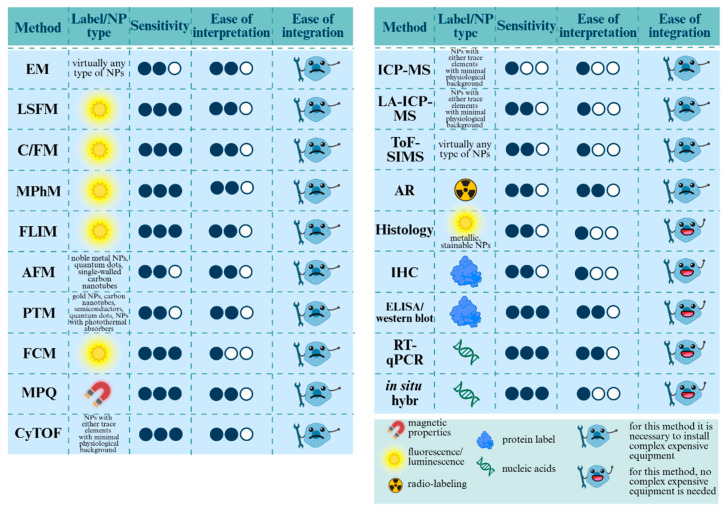
**Comparative advantages of invasive nanoparticle detection techniques in vitro and ex vivo.** The table contrasts five method families: microscopy, cytometry/magnetic readouts, mass spectrometry, autoradiography, and biochemical/histological assays. For each technique it summarizes compatible NP labels, relative sensitivity, ease of interpretation, and ease of integration into workflows. Explanation of abbreviations: electron microscopy (EM), light sheet fluorescence microscopy (LSFM), confocal/fluorescence microscopy (C/FM), multiphoton microscopy (MPhM), fluorescence lifetime imaging microscopy (FLIM), atomic force microscopy (AFM), photothermal microscopy (PTM), flow cytometry (FCM), magnetic particle quantification (MPQ), cytometry by time-of-flight (CyTOF), inductively coupled plasma mass spectrometry (ICP-MS), laser ablation inductively coupled plasma mass spectrometry (LA-ICP-MS), time-of-flight secondary ion mass spectrometry (ToF-SIMS), autoradiography (AR), immunohistochemistry (IHC), in situ hybridization (in situ hybr). In the sections “ease of integration” and “ease of interpretation” metrics reflect the prospective potential of these invasive modalities to be further developed and translated into clinical practice. Created in BioRender. Shipunova, V. (2025) https://BioRender.com/b7ulm5r.

The present section reviews invasive techniques for nanoparticle detection in biological samples, encompassing microscopy, cytometry, histology, and mass spectrometry (Figure 18). These techniques are indispensable for elucidating the mechanisms of nanoparticle internalization, subcellular localization and quantitative elemental or molecular readouts; however, their application necessitates sample destruction. Despite their indispensable role in foundational research, the clinical application of these methods is constrained, with only a minority under evaluation in clinical trials.

### 3.1. Microscopy-Based Methods

#### 3.1.1. Electron Microscopy for Nanoparticle Visualization

Electron microscopy (EM) is one of the most powerful tools for visualizing nanoparticles in tissues and cells. Unlike light microscopy, EM employs accelerated electron beams with picometer-scale wavelengths, providing spatial resolution down to 1–10 nm and enabling the study of ultrastructural details of cells, tissues, and nanomaterials [220].

The two main modalities are transmission electron microscopy (TEM) and scanning electron microscopy (SEM). In TEM, electrons pass through ultrathin sections to reveal internal structures [221,222], whereas SEM scans the sample surface with a focused beam, collecting secondary or backscattered electrons to form an image [223]. Modern instruments operate under high vacuum and include electron guns, lenses, and dedicated detectors [224]. SEM typically achieves resolutions of 1–10 nm with magnifications up to 10^6^ [225,226], while TEM can reach 0.05–0.2 nm resolution with magnifications up to 10^7^ [221,227]. Both techniques are widely used in nanomedicine: SEM is especially valuable for studying interactions of nanoparticles with cell membranes [228,229], and TEM reveals fine spatial relationships between nanoparticles and intracellular structures.

Despite their power, EM methods require complex and time-consuming sample preparation, which can affect resolution [230]. For SEM, samples often need to be dehydrated and coated with a thin metal layer such as gold, silver, or platinum [231]. Critical point drying minimizes artifacts but is costly [232], while hexamethyldisilazane offers a cheaper, though toxic, alternative [233]. Simpler methods, like air-drying, may introduce shrinkage artifacts [234]. Culturing cells directly on substrates before fixation and dehydration can reduce these issues [235]. For example, Goldstein et al. visualized gold nanoparticles in RAW 264.7 macrophages and HaCaT keratinocytes without metal coating [236].

TEM preparation also impacts image quality. Embedding samples in resin can introduce background noise, while cryofixation and Tokuyasu cryosectioning improve preservation but may reduce contrast [237,238]. Nevertheless, TEM still offers unmatched resolution for direct visualization of nanoparticles and cellular components.

**Figure 19:**
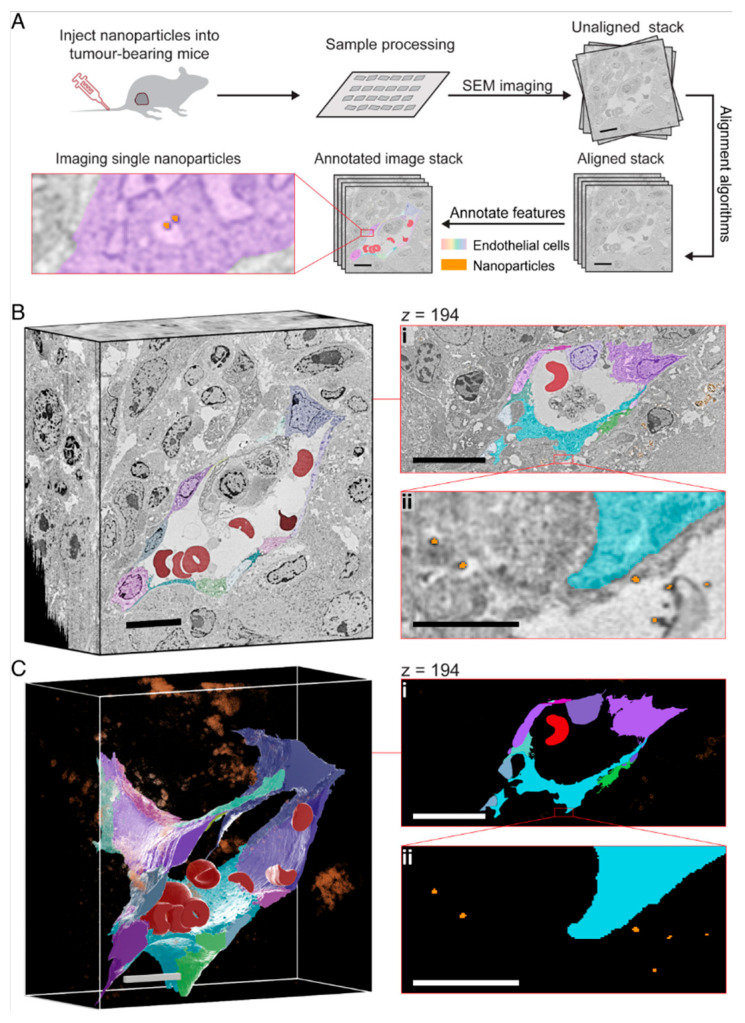
**3D reconstruction of nanoparticle distribution in tumor vasculature.** (**A**) Experimental workflow: tumor-bearing mice were injected with gold nanoparticles, tumors were excised, fixed, embedded, sectioned (70 nm), and mounted on silicon wafers for SEM imaging and annotation of endothelial cells and nanoparticles. (**B**) 3D reconstruction of stacked 2D images with annotated endothelial cells; some cells were removed to visualize vasculature. Insets: (i) 2D slice at the 194th z-plane; (ii) magnified fragment. (**C**) 3D model of annotated endothelial cells (colored) and nanoparticles (orange). Insets: (i) 2D slice at the 194th z-plane; (ii) magnified fragment. Scale bars: 10 µm (1 µm in insets). Reproduced from [242], CC BY-NC-ND.

Practical applications in nanomedicine include particle characterization and studies of cellular uptake. For example, Zelepukin et al. used SEM to demonstrate interactions of magnetic nanoparticles with erythrocytes during circulation time analysis [13]. Sindhwani et al. employed EM to visualize nanoparticle transport across endothelial cells in solid tumors [239]. More recently, Mladjenovic et al. reconstructed 3D maps of nanoparticle distribution in breast tumor vasculature using serial SEM imaging combined with machine learning, showing that 2D EM may miss up to 75% of morphological detail. The experimental workflow and reconstructed models are shown in Figure 19.

In summary, electron microscopy provides unmatched resolution for nanoparticle research but comes with high equipment costs, labor-intensive preparation, and the requirement for ultrathin samples. Nevertheless, it remains indispensable for elucidating how nanoparticles interact with biological systems.

#### 3.1.2. Confocal and Fluorescence Microscopy in Nanoparticle Research

Confocal and fluorescence microscopy belong to the family of optical imaging techniques. Their main advantage is the ability to visualize biological processes in real time and in situ with minimal sample disruption. Fluorescence itself relies on photon absorption by a fluorophore followed by emission of a lower-energy photon within nanoseconds, leading to the characteristic Stokes shift [240]. Compared to conventional one-photon fluorescence microscopy, confocal microscopy offers higher resolution. While the typical resolution of standard fluorescence imaging is 200–300 nm in the XY plane and 500–800 nm along Z, confocal microscopy improves this to ~180–250 nm in XY and 500–700 nm in Z by reducing the illumination spot and rejecting out-of-focus light using a pinhole aperture [241].

For nanoparticle research, however, this resolution is often insufficient. To overcome the diffraction limit, super-resolution fluorescence microscopy has been developed, including methods such as stimulated emission depletion (STED), structured illumination microscopy (SIM), stochastic optical reconstruction microscopy (STORM) and photoactivated localization microscopy (PALM). STED microscopy, for instance, employs two lasers—one to excite fluorophores and another to deplete fluorescence at the periphery thereby narrowing the emission zone and reaching spatial resolutions of 20–40 nm depending on the system [243,244,245].

All fluorescence-based techniques, including confocal microscopy, are suitable for both in vitro and in vivo studies. They require little sample preparation and can even be applied to live specimens [246]. Their limitations are also clear: resolution remains lower than that of electron microscopy, and only fluorescent nanoparticles or labeled nanomaterials can be studied [247,248].

**Figure 20:**
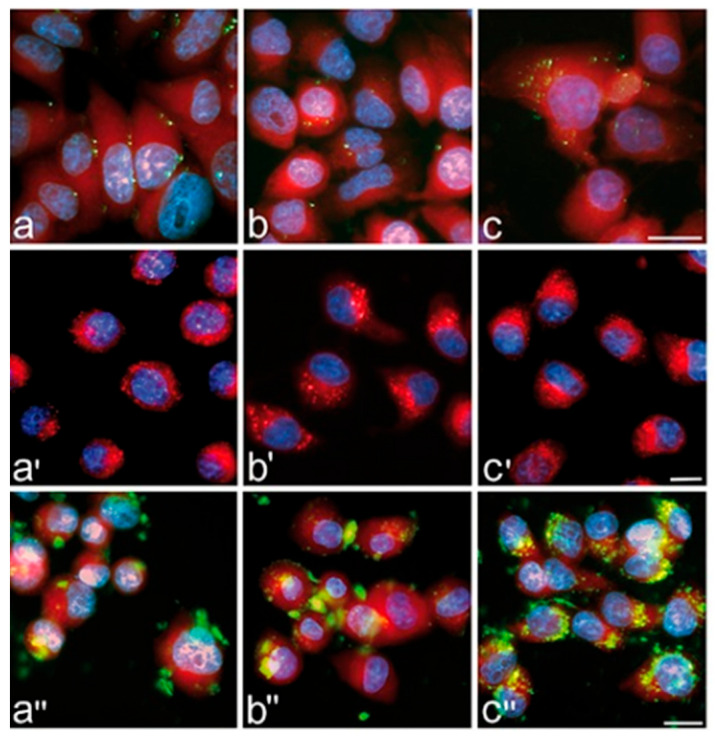
**Nanoparticle accumulation and perinuclear localization in cells.** Intracellular distribution of liposomes (green fluorescence, (**a**–**c**)), polymeric nanoparticles (red, (**a′**–**c′**)), and MSN (green, (**a″**–**b″**)) after 2 h (**a**–**a″**), 24 h (**b**–**b″**), and 48 h (**c**–**c″**) of incubation. All nanoparticles localize in the cytoplasm, mainly perinuclear, without nuclear entry. Polymeric nanoparticles and MSN accumulate over time, whereas liposomes show no clear accumulation. DNA stained with Hoechst 33342 (blue); cytoplasm in (**a**–**c**) and (**a″**–**b″**) with trypan blue (red). Scale bar: 20 μm. Reproduced from [249], CC BY-NC 4.0.

Despite these drawbacks, fluorescence microscopy is widely used in nanotechnology. In vitro, it has proven valuable for tracking nanoparticle internalization and interactions with organelles [249] (Figure 20). It also supports studies of nucleic acid delivery using nanoparticle carriers [250]. Importantly, confocal imaging has been applied beyond cell culture: Reimold et al., for example, visualized the brain distribution of poly(butyl cyanoacrylate) nanoparticles, showing significant accumulation in the central nervous system [251]. Confocal microscopy is now also used directly in vivo, as illustrated by Zhukova et al., who applied intraretinal confocal neuroimaging to track PLGA nanoparticles [252]. Another promising approach is fluorescence imaging in the second near-infrared window (NIR-II, 1000–1700 nm), which allows deep tissue penetration; its effectiveness was recently demonstrated in visualizing single neutrophils in the mouse brain [253].

#### 3.1.3. Multiphoton Microscopy (2P/3P) for Nanoparticle Imaging

Multiphoton microscopy is a form of laser scanning microscopy. It relies on the nonlinear interaction of light with the specimen, in which the probe simultaneously absorbs two or three photons [254]. Longer-wavelength photons are used, as they scatter less and permit deeper tissue penetration (up to 1 mm) compared with conventional confocal microscopy [246].

Multiphoton microscopes typically employ tunable titanium–sapphire lasers as powerful light sources to generate infrared excitation light. The excitation beam passes through a dichroic mirror and an objective lens before reaching the specimen. A dichroic mirror selectively reflects certain wavelengths while transmitting others, directing the emitted light back through the objective toward one or multiple detectors.

This technique can detect both fluorescently modified [255] and unlabeled nanoparticles [256], in both in vitro [257] and in vivo studies [258]. Thus, Priwitaningrum et al. used two-photon microscopy to analyze the penetration of Cy3-labeled polymeric micelles (Cy3-PMC) and polymeric nanoparticles (Cy3-PNP) into three-dimensional tumor spheroids [259]. 4T1 homospheroids and heterospheroids were incubated with these nanoparticles for 24 h before imaging. Cross-sectional images revealed that in homospheroids, both Cy3-PMC and Cy3-PNP penetrated extensively, with fluorescence visible throughout, including the core. In contrast, heterospheroids showed fluorescence mainly at the periphery, likely due to dense collagen acting as a barrier. Cy3-PMC penetrated better than Cy3-PNP, reflecting its smaller size (~70 nm vs. 150 nm). Positively charged NP, regardless of size, adhered to peripheral cells and showed limited penetration into the spheroid interior. This application highlights the utility of multiphoton microscopy for real-time, high-resolution imaging of NP distribution in complex 3D tumor models.

**Figure 21:**
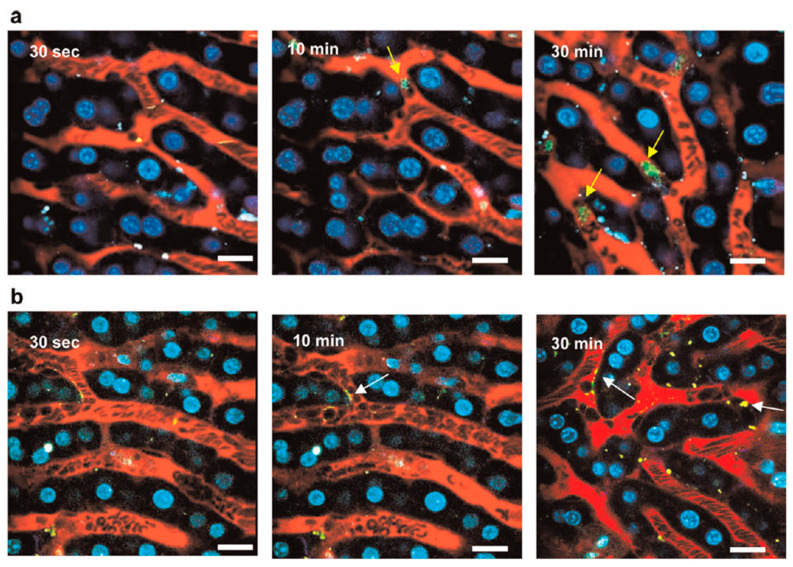
**Multiphoton microscopy imaging of hepatic NP metabolism.** Visualization of the differential hepatic accumulation of (**a**) negatively charged (yellow) and (**b**) positively charged (white) FITC-labeled MSNs approximately 30 μm below the capsule. Red: rhodamine/dextran. Green: fluorescence of FITC-MSNs. Blue: hepatocyte nuclei labeled with Hoechst 33342. Time counted from the moment of injection of MSNs. Scale bars, 50 μm. Reproduced with permission from [42] © American Chemical Society (2012).

Multiphoton microscopy has also been applied for real-time in vivo nanoparticle tracking [42]. MCM-41 mesoporous silica nanoparticles (MSNs), fluorescently labeled with fluorescein isothiocyanate (FITC), were excited in a two-photon process. A mouse was surgically implanted with a hepatic imaging chamber, and FITC-labeled MSNs were injected intravenously via the jugular vein. Additionally, rhodamine B isothiocyanate/dextran 70,000 and Hoechst 33342 were injected to visualize sinusoids and nuclei, respectively. This setup enabled dynamic monitoring of hepatic NP metabolism at subcellular resolution in real time (Figure 21). Compared with traditional confocal microscopy, the use of longer excitation wavelengths minimized photodamage and allowed extended observation periods—critical for real-time visualization of NP distribution in vivo.

Furthermore, multiphoton microscopy has been employed in clinical trials to visualize ZnO NPs in human skin [260,261]. The detection limit of the method is 0.08 fg/µm^3^.

Overall, multiphoton microscopy represents a transformative technology for deep-tissue fluorescence imaging. Its optical sectioning arises from nonlinear excitation confined to the focal volume, reducing out-of-focus photobleaching and phototoxicity and enabling long-term longitudinal studies in living animals. Limitations include high equipment costs, system complexity, slightly lower spatial resolution than confocal microscopy due to longer excitation wavelengths, and the risk of nonlinear photodamage from high-intensity pulsed lasers.

#### 3.1.4. Fluorescence Lifetime Imaging Microscopy

Fluorescence Lifetime Imaging Microscopy (FLIM) is a variation of confocal microscopy based on measuring fluorescence lifetimes. In conventional fluorescence microscopy, signal intensity is the key parameter, while in FLIM the critical information comes from how long a molecule continues to emit light after laser excitation. This property enables FLIM to distinguish target molecules, such as fluorescently labeled nanocarriers, against the background of tissue autofluorescence [262]. FLIM can be implemented in both single-photon confocal and multiphoton microscopy setups.

Fluorescence lifetime depends not only on the intrinsic properties of a fluorophore but also on its local environment—for instance, viscosity, pH, refractive index, or molecular interactions [263]. Changes in fluorescence lifetime can also arise from resonance energy transfer effects such as FRET [260]. This makes FLIM a valuable method for studying cellular metabolism and viability. For example, intracellular levels of NADH and FAD can be quantified using this approach [264,265].

In nanotechnology, FLIM offers distinct advantages over conventional fluorescence imaging. Typically, nanoparticles exhibit longer fluorescence lifetimes than tissue autofluorescence, making them easier to resolve in complex biological environments [266]. One specific example is the work of Luo Gu and colleagues, who used porous silicon nanoparticles excited in the near-infrared range. These biocompatible particles displayed emission lifetimes of 5–13 μs, which is much longer than tissue autofluorescence (<10 ns), resulting in over 50-fold contrast enhancement in vitro and more than 20-fold in vivo [267]. Even higher contrast was achieved in the study by Jinmyoung Joo et al., where porous silicon nanoparticles, including iRGD-peptide–modified variants, produced more than a 100-fold improvement [268]. The authors compared imaging with a standard IVIS system, CW imaging, and GLISiN under LED and pulsed laser excitation, finding the best results with GLISiN and pulsed excitation.

Although porous silicon nanoparticles are most commonly used in FLIM, other nanomaterials have also been explored, including lanthanide-doped nanoparticles [269] and gold clusters or quantum dots [270].

Overall, FLIM provides a powerful means of visualizing nanoparticles by exploiting fluorescence lifetime differences. However, the method faces practical challenges: it requires expensive equipment with ultrafast pulsed lasers and photon-counting detectors, its imaging depth is limited to a few hundred microns due to scattering and absorption, and it is not yet applied in clinical practice. Importantly, successful application of FLIM depends on using bright, photostable nanoparticles.

#### 3.1.5. Atomic Force Microscopy

Atomic force microscopy (AFM) is a high-resolution scanning probe technique that generates three-dimensional topographies by measuring the forces between a sharp tip and a sample surface, enabling the quantification of surface roughness at the atomic scale [271]. AFM achieves extremely high resolution, with capabilities down to the atomic level (0.1 nm horizontal and 0.01 nm vertical) [272].

AFM is widely used for characterizing the shape and size of nanoparticles [273], but it can also be applied to nanoparticle-cell interactions [274] and the biodistribution of nanoparticles in tissues [275].

**Figure 22:**
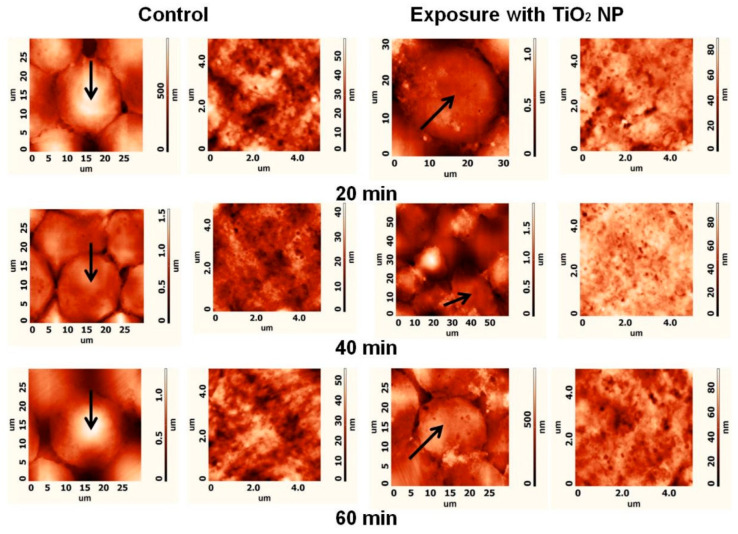
**Representative AFM images of CHO control cells (left) and cells after 20-, 40-, or 60-min exposure to TiO_2_ (right)**. The second column shows the ultrastructure of the cell plasma membrane (5 × 5 µm). Reproduced from [276], CC BY.

D. Batiuskaite et al. [276] investigated the internalization of nanoparticles into ovarian cancer cells using AFM. Figure 22 shows the topography of the CHO cell membrane. The images revealed pits identified as caveolae, characterized by surface height differences and implicated in the endocytosis of TiO_2_ nanoparticles.

A major advantage of AFM is its ability to image samples under native conditions without staining, labeling, or fixation, while providing a high signal-to-noise ratio and simultaneously assessing multiple physical properties. However, its primary limitation is that measurements are restricted to surfaces, limiting access to subsurface or internal structures.

#### 3.1.6. Photothermal Microscopy

The core principle of photothermal microscopy is the absorption of light (the heating beam) by nanoparticles, which enables visualization without the need for fluorescent labeling. Noble metal nanoparticles, quantum dots, and single-walled carbon nanotubes can all be detected using this method. The strength of the photothermal signal is directly proportional to the nanoparticle’s absorption cross-section [277]. This technique is highly sensitive and can detect particles with absorption cross-sections as low as 10^−15^ cm^2^ (e.g., 2 nm gold nanoparticles) [278].

**Figure 23:**
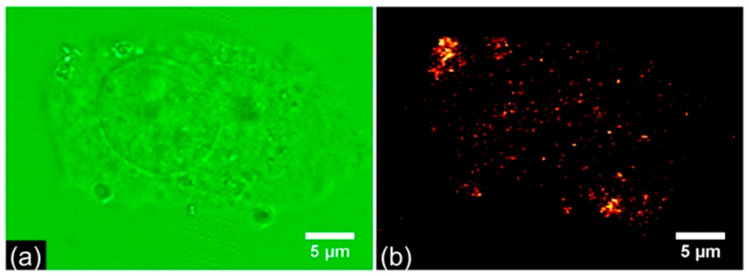
**Comparison of wide-field (a) and photothermal (b) microscopy images of a HeLa cell with PLL-coated 20 nm BaTiO_3_ nanoparticles.** The 3D photothermal reconstruction (from 2 μm interval optical sections) visualizes the intracellular distribution of non-fluorescent particles. Reproduced from [280], CC BY 4.0.

Photothermal microscopy offers high spatial resolution (down to 50 nm) and allows real-time monitoring of nanoparticle-cell interactions [279]. It is currently used to track single nanoparticles in live cells [280,281,282] and to detect proteins in cells using nanoparticles [283] (Figure 23).

A major advantage of photothermal microscopy in bioimaging is its label-free operation, enabling visualization of non-fluorescent nanoparticles. Because the detected objects are not susceptible to photobleaching, this technique permits prolonged, stable observation of processes such as nanoparticle uptake and intracellular transport. However, its application is limited by dependence on the photothermal conversion efficiency of the nanomaterial, restricting use to strongly absorbing agents. In addition, the need for a high-intensity heating laser raises concerns about potential photothermal damage to delicate biological specimens, which must be carefully managed to maintain sample viability.

### 3.2. Cytometry-Based Methods

#### 3.2.1. Flow Cytometry

Flow cytometry (FCM) is a powerful, rapid analytical technique for quantitative and qualitative high-throughput characterization of cells and particles based on their optical properties. In nanotechnology, FCM enables measurement of nanoparticle accumulation in individual cells.

In this method, a population of cells labeled with fluorescently tagged nanoparticles is directed into a high-speed laminar flow (up to 20 m/s), which is focused into a single stream. Laser excitation then induces fluorescence emission, while forward and side scattering are detected using photodetector arrays equipped with spectral filters. Forward scatter (FSC) correlates with cell size, while side scatter (SSC) reflects intracellular complexity, such as granularity and nuclear morphology. Specifically, the fluorescent signal from labeled nanoparticles provides a direct measure of their presence within or on individual cells. Detectors convert scattered and fluorescent signals into digital data for cell population identification, quantification, and fluorescence intensity analysis, thereby enabling determination of nanoparticle uptake efficiency.

FCM can detect both fluorescently labeled and non-fluorescent nanoparticles. Labeled nanoparticles are measured through their emitted fluorescence, while non-fluorescent particles—such as silver, titanium dioxide, zinc, gold, and silica nanoparticles—can be detected by analyzing SSC signals [284,285,286]. The intensity of the SSC signal depends on particle size and refractive index, with larger or more refractive particles producing stronger signals.

The sensitivity of FCM depends on fluorophore brightness, while for nanoparticles detected SSC, particle size is critical. For example, in a study using nanoparticles labeled with Alexa Fluor 488, the LOD was estimated at nine fluorophore molecules per nanoparticle [287]. For polystyrene particles, detection limits were calculated as low as 23 particles per cell for 500 nm particles, and ~8000 particles per cell for smaller 50 nm particles [288].

Recent advances in flow cytometry for nanoparticle analysis aim to enhance resolution and reduce noise. These improvements include optimized optics, differential signal amplification, specialized violet lasers, and image-coupled systems [289,290,291]. Such advancements improve the detection and characterization of sub-micron particles, enabling researchers to better quantify and differentiate nanoparticles.

Traditionally, FCM is employed in vitro to analyze nanoparticle-cell interactions, providing insights into cellular uptake, targeting specificity, and toxicity. While its application in nanomedicine remains largely experimental and preclinical, FCM is FDA-approved for diagnostic use in hematology and immunology [292]. In nanoparticle studies, ex vivo FCM is applied to biological samples such as blood and dissociated tissue [293,294,295]. For instance, Piotrowski-Daspit et al. used FCM to analyze systemic delivery of poly(amine-co-ester) nanoparticles in mice, revealing cell-type- and tissue-specific accumulation patterns in blood and organs [293]. Similarly, Korangath et al. employed FCM to characterize immune cell alterations in huHER2 allograft tumors following nanoparticle or free antibody treatment, reporting a decrease in total live cells and a significant reduction in CD45+ immune cells within tumors 24 h post-injection [296].

In summary, FCM is a highly sensitive and rapid method for analyzing nanoparticle-cell interactions both in vitro and in vivo. Ex vivo FCM enables high-throughput single-cell analysis of nanoparticle uptake, distribution, and targeting efficiency, with advantages such as speed, multiplexing capability, and compatibility with diverse nanoparticle types, primarily fluorescent. However, it requires blood or tissue extraction and complex sample preparation, and does not provide dynamic data, limiting its application in real-time pharmacokinetics. As described in the previous section, in vivo flow cytometry extends these capabilities to living organisms, allowing noninvasive, real-time monitoring of fluorescent nanoparticles in the bloodstream without sample collection. Nevertheless, IVFC is limited by shallow detection depth, restricted spatial resolution, and reliance on fluorescent labeling. Despite these challenges, both in vivo and ex vivo FCM remain valuable tools for investigating nanoparticle-cell interactions.

#### 3.2.2. MPQ-Cytometry

Fluorescence-based detection methods are widely employed to evaluate the biodistribution and accumulation of nanoparticles in target organs. However, their use for precise quantitative assessment faces significant methodological challenges, including tissue autofluorescence, the dependence of signal intensity on probe depth, and fluorescent dye quenching, among others [58,297,298]. In contrast, the unique advantage of the MPQ method lies in its ability to provide highly sensitive in vivo quantitative assessment of magnetic label accumulation in target organs, free from background noise generated by biological tissues. The operational principle of the MPQ technique is described in Section 2.1.2. This approach involves quantifying the magnetic signal of extracted organs by placing them within the detector’s measuring coil. The obtained signal is then normalized to the total magnetic signal from all harvested organs, allowing quantitative determination of the percentage of the administered nanoparticle dose accumulated in a target organ.

The MPQ method finds significant application in oncological research, particularly for assessing nanoparticle behavior in tumors. For example, it has been used to study how external magnetic fields affect the selective accumulation of nanoparticles in tumors [58]. Among recent works, Kolesnikova et al. used the MPQ method to evaluate the specificity of accumulation for two types of targeted nanoparticles in mice tumors in vivo [44]. Its applicability further extends to other in vivo applications, such as the development of an isolated perfused liver model to study nanoparticle behavior within the hepatic vasculature [299], and investigating the biodegradation profile of magnetic nanoparticles in organisms [68]. Thus, MPQ provides an excellent signal-to-noise ratio and enables quantitative assessment of magnetically labeled nanoparticle accumulation in organs, while avoiding the pitfalls associated with fluorescence-based detection methods. A primary limitation of this technique is the requirement for magnetic materials, combined with the fact that quantification is generally feasible only ex vivo. An exception is represented by small laboratory rodents, where the tail can be placed inside the detector coil, enabling limited in vivo measurements.

#### 3.2.3. Cytometry by Time-of-Flight

CyTOF, or mass cytometry, represents an advanced hybrid technique combining principles of flow cytometry and inductively coupled plasma time-of-flight mass spectrometry (ICP-TOF-MS). First developed by Bandura et al. in 2009 [300], this method enables simultaneous analysis of up to 50 distinct cellular markers with high specificity. In this approach, cell suspensions are incubated with antibodies conjugated to stable metal isotopes, allowing precise detection of antibody-cell interactions. Similar to conventional inductively coupled plasma mass spectrometry, detection occurs through measurement of the mass-to-charge (*m*/*z*) ratios of the metal-tagged antibodies, providing exceptional multiplexing capability.

The methodology operates as follows: a cell suspension with bound antibodies (or other probes) is introduced into a nebulizer that generates single-cell droplets. These droplets are transported to the inductively coupled plasma (ICP) source, where sample ionization occurs (see ICP-MS section for details), followed by detection of the bound probes. The system employs a time-of-flight (TOF) mass analyzer that simultaneously detects ions based on their *m*/*z*-dependent velocities, as determined by their flight times. Compared to conventional flow cytometry, CyTOF offers significant advantages by enabling simultaneous measurement of substantially more parameters per single cell (currently up to 50 parameters, with a theoretical capacity exceeding 100 markers), primarily due to minimal signal spillover between metal channels [300,301]. This technological advancement has considerably expanded cellular analysis capabilities, creating new opportunities for high-dimensional single-cell profiling [302,303].

**Figure 24:**
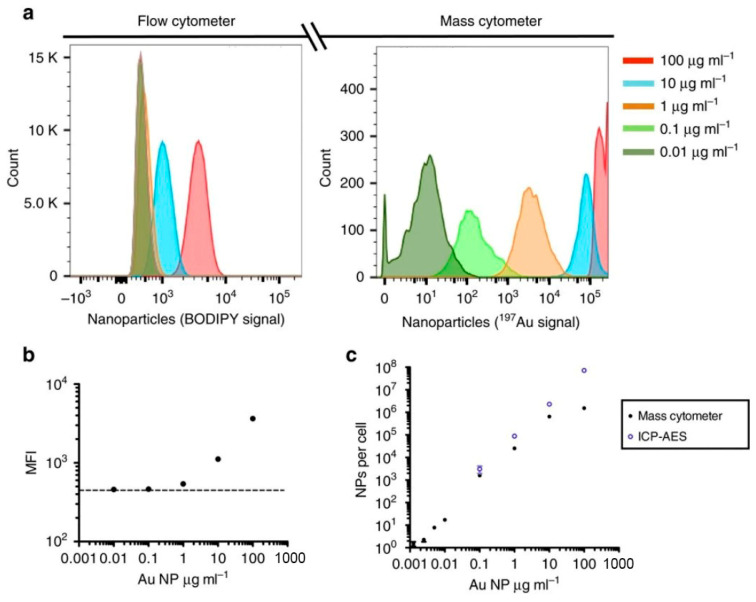
**Sensitive detection of gold nanoparticles in single cells with a wide dynamic range by CyTOF.** (**a**) Histogram of gold NP levels in cells treated at five different concentrations. (**b**) Median fluorescence intensity (MFI) measured by flow cytometry at the same concentrations. (**c**) Quantitative analysis of gold NPper cell by mass cytometry compared with parallel bulk measurements of gold NP uptake by ICP atomic emission spectrometry. Reproduced from [304], CC BY 4.0.

Similar to ICP-MS, CyTOF has been adapted for quantitative analysis of metal nanoparticles in biological systems. Yang et al. demonstrated detection of 3 nm gold nanoparticles at levels as low as 10 nanoparticles per sample, with an upper detection limit of approximately 1.5 × 10^6^ particles per cell, an improvement of ~2400-fold over conventional flow cytometry [304] (Figure 24).

This technique has become instrumental for both in vitro and in vivo studies of nanoparticle-cell interactions, particularly when combined with antibody-based phenotyping, enabling precise identification of specific cell types involved in nanoparticle uptake and distribution [305,306].

In summary, CyTOF represents an advanced analytical platform that synergizes the high-throughput capabilities of flow cytometry with the exceptional sensitivity and multiplexing power of ICP-MS. The combination of multiplexed cellular marker analysis and nanoparticle quantification in a single platform represents a major methodological advancement for single-cell studies of nanomaterial–biological interactions. Its unique advantages, including minimal signal overlap, broad dynamic range, and compatibility with both antibody-based phenotyping and nanoparticle tracking, have established it as an indispensable tool for advanced research in immunology, nanomedicine, and toxicology, opening new horizons in multiparametric single-cell analysis and nanomaterial characterization.

### 3.3. Mass Spectrometry-Based Methods

#### 3.3.1. Inductively Coupled Plasma Mass Spectrometry

ICP-MS is a highly sensitive quantitative technique for multi-elemental analysis at ultratrace levels, first commercialized in 1983. The method detects positively charged ions by their mass-to-charge ratio (*m*/*z*), enabling not only trace element quantification but also isotopic analysis [307,308,309].

The principle is straightforward: the liquid sample is nebulized and introduced into the ICP source, where high temperatures dissociate it into atoms and ionize them. The resulting positively charged ions are extracted and separated according to their *m*/*z*. The ICP itself is generated from argon gas flowing through a quartz torch placed within an induction coil. A radiofrequency alternating current energizes the coil, while a Tesla coil initiates an electrical spark, producing Ar^+^ ions and electrons. These electrons are confined by a magnetic field and accelerated in circular trajectories. Their collisions with neutral argon atoms trigger further ionization, sustaining the plasma. This cascade provides the extreme thermal environment (6000–10,000 K) required for efficient ionization of a broad range of elements [310,311,312]. ICP-MS achieves detection limits from 1 to 100 ng/L (depending on the element) and combines sensitivity with multi-element and isotopic analysis, making it indispensable across fields such as environmental monitoring, archaeology, forensics, and biomedicine [307,311].

In nanomedicine, ICP-MS is widely used to study nanoparticle biodistribution. Non-invasive applications include monitoring excretion kinetics urine and feces, whereas invasive approaches (blood and organ homogenates) provide detailed pharmacokinetics, such as circulation half-life and tissue-specific accumulation [253,313,314,315,316]. The trade-off is that sample digestion required for analysis destroys tissue architecture, sacrificing spatial information.

**Figure 25:**
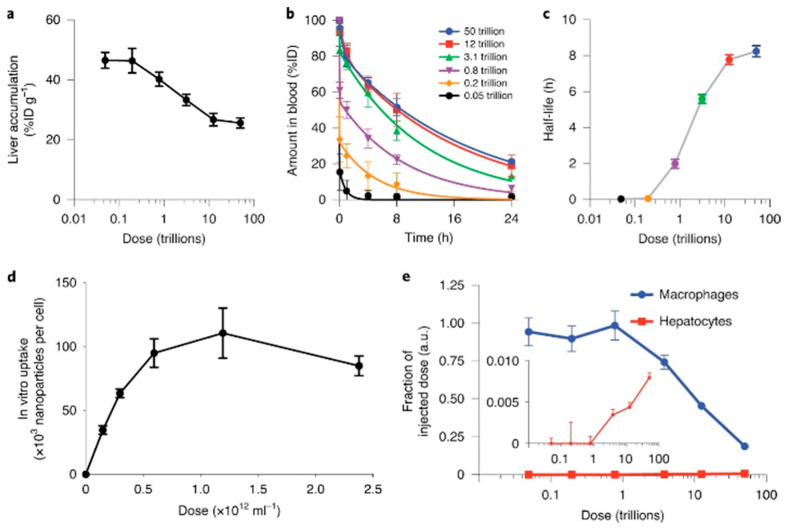
**Biodistribution of gold nanoparticles measured by ICP-MS.** (**a**) Liver accumulation of gold nanoparticles 24 h post-injection, determined using ICP-MS. (**b**) Nanoparticle blood kinetics. (**c**) Half-life of nanoparticles. (**d**) In vitro macrophage uptake over 24 h. (**e**) Quantification of the nanoparticle signal in liver F4/80+ Kupffer cell macrophages and autofluorescent+ hepatocytes using histology. Reproduced with permission from [321] © Springer Nature (2020).

For reliable detection, nanoparticles should contain elements with minimal biological background (e.g., lanthanides) or isotopic labels of endogenous elements. This is why ICP-MS is most often used for metal nanoparticles (Figure 25), which provide superior signal-to-noise compared to organic nanomaterials. Iron oxide nanoparticles, for example, require isotopic enrichment or lanthanide doping to overcome interference from natural iron, whereas rare earth–containing particles can be tracked at ultratrace levels [317]. For example, Crayton et al. demonstrated that lanthanide-doped iron oxide nanoparticles could be detected in blood and organs at parts-per-billion concentrations [318], while regular SPIONs remain challenging to detect due to background iron (detection limit ~0.4 g/L, practical ~0.1 g/L) [319,320].

A breakthrough came in 1986 with the introduction of single-particle ICP-MS (SP-ICP-MS). By diluting samples to <10^8^ particles/L, each signal pulse corresponds to a discrete nanoparticle, enabling simultaneous quantification of nanoparticle number (ppt levels), size distribution, and elemental composition [322,323,324]. SP-ICP-MS has since been applied to food, environmental, and biological matrices, and is particularly valuable for studying nanoparticle agglomeration and stability [324,325,326,327].

In summary, ICP-MS has become a cornerstone for nanoparticle detection and characterization, offering exceptional sensitivity down to single particles. Its limitations include matrix interferences and spectral overlaps, where isobaric elements and polyatomic ions complicate accurate quantification [307]. These challenges highlight the need for advanced correction strategies or complementary methods to ensure robust interpretation in complex biological samples.

#### 3.3.2. Laser Ablation Inductively Coupled Plasma Mass Spectrometry

While ICP-MS enables highly accurate quantification of elemental and isotopic composition in biological samples, providing precise measurements of nanoparticle content, this method cannot determine nanoparticle localization due to its destructive sample preparation requirement (acid digestion and homogenization). Spatial mapping of nanoparticles, however, is essential for understanding their biological impact, therapeutic efficacy, and potential side effects. To address this limitation, LA-ICP-MS has been developed as an alternative approach. In LA-ICP-MS, intact frozen or paraffin-embedded tissue sections (20–200 μm thick) are subjected to localized laser ablation, where a focused beam vaporizes microscopic regions of interest. The ablated particles are transported via gas flow to the ICP-MS, where they undergo atomization, ionization, and analysis (see Section ICP-MS for details). This technique preserves spatial information while maintaining the quantitative capabilities of conventional ICP-MS [328,329,330].

LA-ICP-MS has been widely employed in fundamental research for elemental mapping and bioimaging in biological specimens, including both animal and plant tissues [330]. While its spatial resolution (10–100 μm for nanosecond lasers and <1 μm for femtosecond lasers) remains lower than that of scanning electron microscopy—primarily due to limitations such as laser beam diameter—it is nevertheless suitable for precise analyses at the cellular scale. LA-ICP-MS shows reduced sensitivity compared to ICP-MS, with detection limits commonly observed between a few ng/g and a few µg/g, depending on analyte characteristics and operational parameters [331,332].

**Figure 26:**
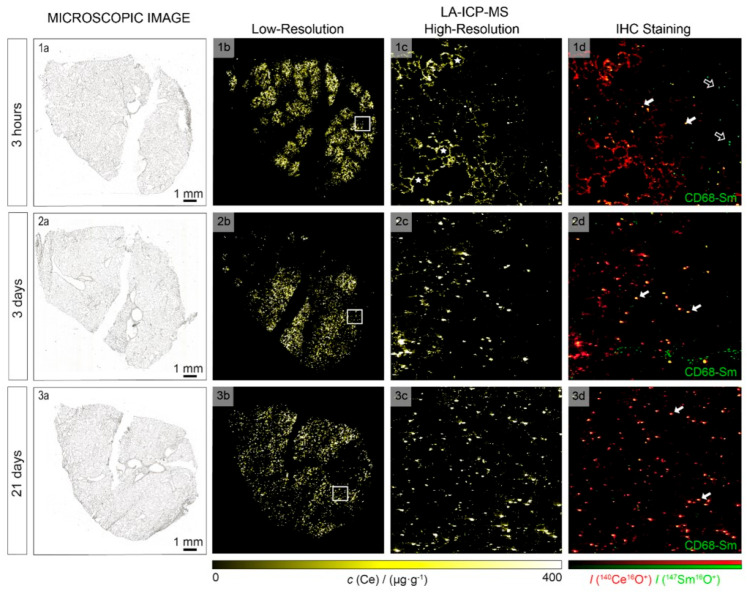
Spatially resolved analysis of the CeO_2_ nanoparticles distribution in the rat lung by means of LA-ICP-MS. Animals received an intratracheal instillation of 0.6 mg of CeO_2_ per lung and were sacrificed after 3 h (1), 3 days (2), and 21 days (3). (**a**) Corresponding bright-field microscopic images. (**b**) Quantitative Ce distribution by means of low-resolution LA-ICP-MS. (**c**) Quantitative Ce distribution by means of high-resolution LA-ICP-MS in the highlighted regions. (**d**) Corresponding overlay of Ce (red) and Sm (green) after IHC staining with anti-rat CD68-Sm. Yellow pixels indicate CD68-positive AM co-localized with Ce. Reproduced with permission from [334] © American Chemical Society (2022).

In nanoparticle research, LA-ICP-MS has proven effective for investigating metal nanoparticle uptake in ecotoxicological model organisms (zebrafish embryos and crustaceans [333]) as well as biodistribution studies in mice, often in combination with immunohistochemical techniques [334,335,336] (Figure 26). The limit of detection in LA-ICP-MS strongly depends on both the natural abundance of the element in biological samples and its inherent ionization efficiency. For trace elements measured in certified reference materials, reported LOD values range from 4 to 670 mg/kg (ppm) [337].

The limitations of LA-ICP-MS are broadly similar to those of conventional ICP-MS, including interference from the biological matrix and spectral overlaps, which can compromise detection accuracy. In addition, the laser ablation process itself introduces uncertainties related to variability in ablation efficiency and transport to the ICP source [330,338].

In conclusion, LA-ICP-MS represents a significant advancement over conventional ICP-MS by preserving spatial information while maintaining quantitative analysis capabilities. Its detection limits and resolution at the cellular range make it particularly valuable for nanoparticle biodistribution studies, despite inherent limitations from matrix effects and ablation-related uncertainties. When combined with complementary imaging techniques, LA-ICP-MS provides researchers with a powerful analytical platform for comprehensive nanoparticle characterization in biological systems.

#### 3.3.3. Time-of-Flight Secondary Ion Mass Spectrometry

Time-of-flight secondary ion mass spectrometry (ToF-SIMS) is one of the most informative techniques for surface analysis. It allows researchers to map the distribution of chemical labels and nanoparticles on sample surfaces, analyze tissues and cells, and perform molecular composition studies [339,340]. This method is often applied to lipid profiling in biological samples (lipidomics) and to metabolic pathway studies (metabolomics) [341]. Recent advances in ToF-SIMS have made it possible to investigate samples in situ, enabling the study of subcellular drug distribution and drug–target interactions [342].

The principle of SIMS lies in detecting secondary ions ejected from a surface by bombardment with a primary ion beam. The high-energy primary ions interact with several atomic layers, causing the emission of secondary ions (SI). These are then analyzed by a time-of-flight mass analyzer, where all ions are given the same kinetic energy per charge, but their velocities differ according to mass. This provides detailed information on the composition and structural characteristics of the surface components [343]. Compared to other surface analysis techniques, SIMS offers notable advantages: it provides high resolution (down to tens of nanometers) and makes it possible to study very small regions, down to the micrometer scale [344]. Moreover, SIMS can detect a wide range of elements and probe chemical bonding information [345,346].

A standard ToF-SIMS instrument includes an ion source, a time-of-flight mass analyzer (a tube up to a meter long), and a high-vacuum chamber. Additional modules include a sample introduction chamber and ion optics for both primary and secondary ions [347]. Such instruments are typically large (several square meters), require stable environmental conditions (temperature, vibration, power supply), and are expensive to operate.

Different types of primary ions can be used. For example, Daniel J. Graham and colleagues demonstrated the efficiency of tri-atomic Bi3^+^ ions on HeLa cells [348], while John S. Fletcher and co-workers applied fullerene cluster ions (C60) to study frog oocytes [349].

**Figure 27:**
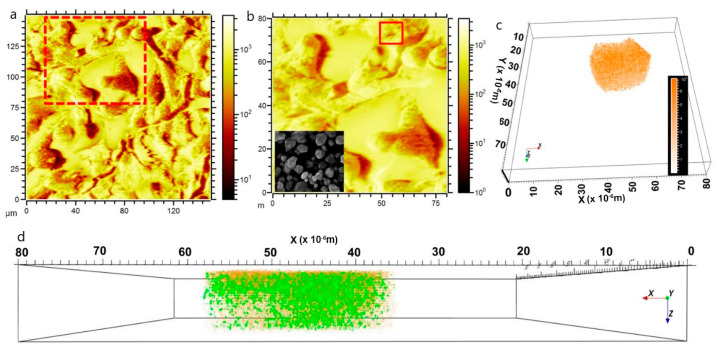
**ToF-SIMS ion imaging of quasi-spherical Au-NP at Au-ion concentrations up to 1 mM.** (**a**,**b**) Topography: summed ion reconstruction of A549 cell surfaces; inset shows SEM of the nanostructure. (**c**) 3D reconstruction of a single cluster with Au^+^ (*m*/*z* 196.97) signals. (**d**) Enlarged depth-resolved reconstruction showing lateral distribution of Au^+^ (orange) and threonine-O-3-aurate phosphate (green). Reproduced from [350], CC BY 4.0.

ToF-SIMS has become especially valuable in nanobiotechnology and nanotoxicology. It allows nanoparticle mapping within cells and assessment of their effects on surrounding tissues. For example, A. V. Singh and co-workers showed how gold nanoparticles undergo biomineralization, with particle shape depending on gold concentration and seed addition [350]. The method also enables 3D label-free reconstructions of individual cells (Figure 27). Andrea Haase and colleagues used laser SIMS/ToF-SIMS to study silver nanoparticle distribution in macrophages, revealing intracellular aggregates and small clusters on the outer membrane, as well as oxidative stress effects on plasma membranes [351]. Another example comes from P.-L. Lee, who applied ToF-SIMS to ZnO nanoparticles: changes in calcium-to-potassium ion ratios and levels of phosphocholine and glutathione across different cellular compartments indicated cytotoxic effects as ZnO particles gradually penetrated cells [352]. Cytotoxicity was further confirmed using ZnO nanoparticles labeled with the stable isotope ^68^Zn.

Overall, ToF-SIMS offers several advantages. In addition to high resolution (down to 100 nm), it is surface-sensitive and enables molecular mapping without the need for extra labels or dyes. Sample preparation is straightforward, making it particularly appealing for nanostructure analysis. However, the method also has drawbacks. Bombardment inherently damages the surface, especially in 3D reconstructions. A major limitation is the matrix effect, where signals from certain compounds can be enhanced or suppressed depending on their local environment, making the method semi-quantitative. Finally, ToF-SIMS instruments are expensive and space-demanding, requiring dedicated facilities.

### 3.4. Biochemical and Luminescent Methods

#### 3.4.1. Chemiluminescent Analysis

Chemiluminescence (CL), a process in which chemical reactions generate light, is widely applied in chemical detection, bioanalysis, and bioimaging. It is valued for its efficient light emission, unique underlying mechanism, and absence of background noise from photoexcitation [353]. One of the most extensively studied chemiluminescent systems involves the oxidation of luminol, lucigenin, or peroxyoxalate in the presence of hydrogen peroxide (H_2_O_2_) under alkaline conditions. This reaction is typically catalyzed by peroxidase enzymes, such as horseradish peroxidase. In recent years, the concept has been extended to nanotechnology with the discovery that various nanoparticles, termed nanozymes, possess intrinsic enzyme-mimicking activity, particularly peroxidase-like activity. A broad range of nanomaterials have been reported to exhibit such properties, including noble metal nanoparticles (e.g., Au, Ag, Pt), metal oxides (e.g., CuO, Fe_3_O_4_, Co_3_O_4_) [354,355,356,357], and carbon-based nanomaterials [358]. Beyond classical nanozymes, recent advances have demonstrated that self-immolative polymers can also be engineered into chemiluminescent nanoparticles [359].

**Figure 28:**
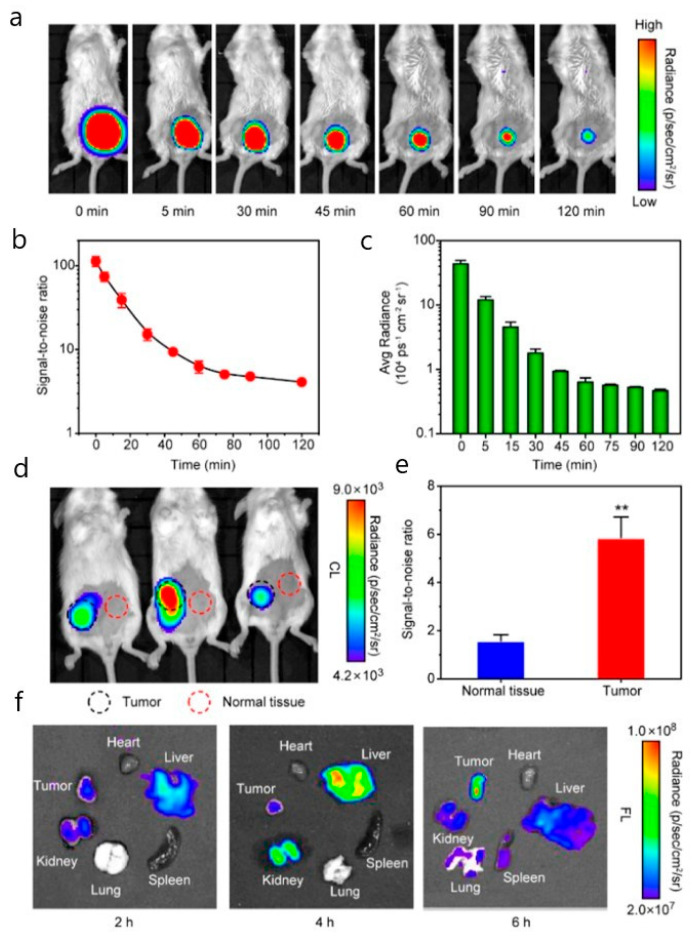
**CL imaging of ROS in mice.** (**a**) In vivo CL imaging in mice s.c. injected with CPPO/BSA@AuNCs and H_2_O_2_. (**b**) SNR analysis of CL. (**c**) Quantitative analysis of CL signals over time. (**d**) In vivo CL images of mice intratumorally injected with CPPO/BSA@AuNCs. (**e**) SNR differences between tumor and normal tissue. ** *p* < 0.01. (**f**) Ex vivo CL images of main organs (heart, liver, spleen, lung, and kidney) and tumors from a mouse bearing a breast tumor after intravenous injection of CPPO/BSA@AuNCs for 2, 4, and 6 h. Reproduced with permission from [360] © American Chemical Society (2024).

For instance, Wang et al. developed CPPO/BSA@AuNCs nanoparticles by encapsulating the oxalate-based chemiluminescent substrate CPPO within bovine serum albumin–capped gold nanoclusters, which exhibited aggregation-induced NIR emission. This platform enabled deep-tissue imaging with penetration depths exceeding 27 mm and allowed accurate visualization of both primary and metastatic breast tumors in mice, outperforming fluorescence and conventional blue CL for tumor detection [360] (Figure 28).

CL-based detection offers several notable advantages: high sensitivity and selectivity, minimal background due to the absence of external excitation, and the ability to perform long-term imaging without overheating or photobleaching. Moreover, it allows the use of nanozymes as label-free probes. Reported LOD in in vivo applications vary depending on the nanozyme type and system but are typically in the nanomolar range [361]. The method is relatively simple, cost-effective, and well-suited for in vivo imaging, particularly in pathological tissues with elevated H_2_O_2_ levels. Nonetheless, its broader application is limited by the requirement for nanozymes with peroxidase-like activity, the potential cytotoxicity of reagents such as luminol, and restricted spatial resolution due to light scattering in tissues.

Although CL assays are widely established in in vitro diagnostics, no chemiluminescent nanoparticle–based platforms are currently approved for either in vitro or in vivo clinical use. Still, their strong potential for in vivo imaging suggests that ongoing advances may soon enable medical translation.

#### 3.4.2. Bioluminescent Methods

Bioluminescence (BL) results from enzymatic reactions in living organisms, most commonly involving luciferase enzymes and their substrates (e.g., luciferin, coelenterazine). Unlike fluorescence, bioluminescence does not require external light excitation, resulting in extremely low background signal and high sensitivity, which makes it particularly advantageous for in vivo imaging [362]. Typically, BL relies on the oxidation of luciferin, catalyzed by luciferase, in the presence of oxygen and cofactors such as ATP or metal ions. This reaction yields oxyluciferin and releases energy in the form of light.

**Figure 29:**
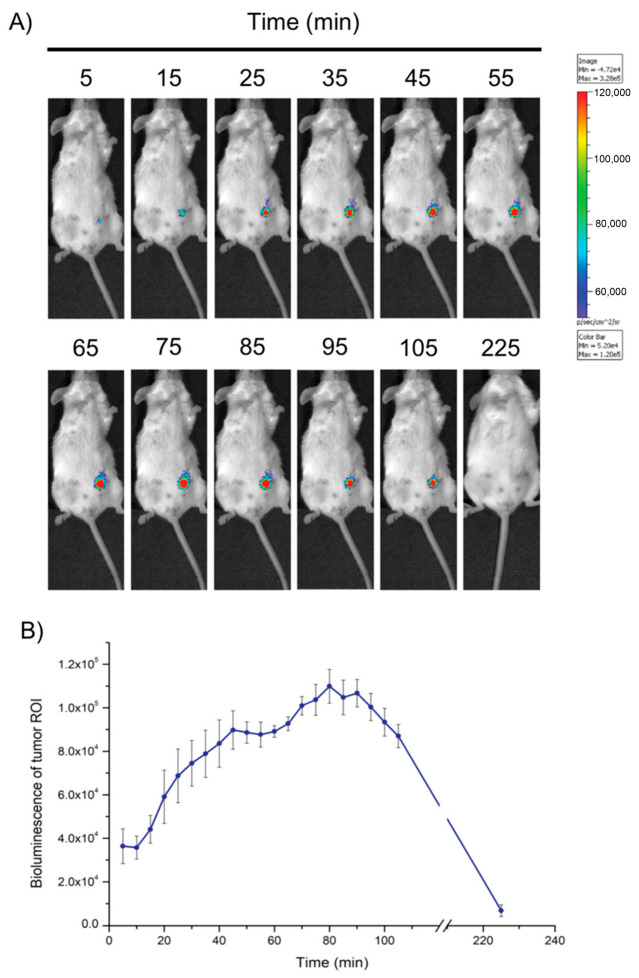
**Bioluminescence imaging of mice with 4T1 tumors.** (**A**) Bioluminescence images of mice i.v. injected with H-ferritin nanoparticles conjugated to D-luciferin. (**B**) Quantification of tumor bioluminescence over time. Reproduced with permission from [363] © John Wiley and Sons (2020).

For in vivo nanoparticle detection, a labeling strategy is essential. Nanoparticles may either carry luciferase directly (through covalent attachment, immobilization, or genetic fusion) or encapsulate its substrate for controlled release. For example, Bellini et al. developed a targeted delivery system based on H-ferritin nanoparticles conjugated to D-luciferin via a disulfide linker that is cleaved in the intracellular reducing environment, releasing the substrate in luciferase-expressing tumor cells and generating a bioluminescent signal. Upon intravenous injection into luciferase-expressing 4T1 tumor-bearing mice, the bioluminescence signal peaked around 80 min and declined thereafter (Figure 29), while free D-luciferin cleared within 40 min [363].

Beyond simple luciferase-conjugated nanoparticles, more advanced nanosystems are being developed based on the bioluminescence resonance energy transfer phenomenon (BRET) to enhance imaging contrast in deep tissues. BRET is a non-radiative energy transfer process similar to Förster resonance energy transfer (FRET), but instead of requiring external light excitation, it uses energy generated by a bioluminescent enzyme reaction. Xiong L. and colleagues were the first to create an imaging system that combined BRET and FRET [364]. They synthesized polymer nanoparticles incorporating a Renilla luciferase mutant (Luc8), a conjugated polymer (MEH-PPV), and an NIR dye (NIR775). Upon oxidation of coelenterazine by Luc8, energy was transferred to MEH-PPV and then to NIR775, resulting in strong NIR emission. In vivo, these nanoparticles functionalized with cRGD peptides enabled high-contrast imaging of glioblastoma xenografts in mice, achieving tumor-to-background bioluminescence ratios >100. Even small tumors (2–3 mm) were clearly detected just 5 min post-injection. The particles also enabled efficient mapping of lymph nodes after intravenous or intradermal administration.

BL is a highly sensitive method for in vivo applications, characterized by a high signal-to-noise ratio, minimal background signals, and excellent temporal and spatial resolution. Although exact LOD in vivo is not reported, nanoparticle/BLI-based assays demonstrate extremely low detection limits in vitro, often reaching the picomolar to femtomolar range [365,366,367]. While not yet approved for clinical use, they are extensively used in preclinical studies for tracking nanoparticle biodistribution and visualizing tumor growth or metastasis in laboratory animals [368,369,370].

BL imaging is therefore a powerful tool for in vivo nanoparticle detection, offering exceptional sensitivity, non-invasiveness, and high imaging contrast. Its synergy with nanomaterials expands its utility in bioimaging, biosensing, and therapy. Limitations include shallow tissue penetration unless using NIR probes, the need for substrate injection, possible luciferase instability or ATP dependence, and rapid signal decay without stabilization. Ongoing development of advanced nanosystems is expected to further enhance the capabilities and broaden the applications of BL in nanomedicine.

### 3.5. Combined and Hybrid Methods

#### Autoradiography

Autoradiography (AR) is based on the detection of ionizing radiation emitted by radionuclides. It relies on the principle that radioactive isotopes undergo spontaneous nuclear decay, emitting particles (α, β^−^, β^+^) or photons with characteristic energies. These emissions can expose a radiation-sensitive medium placed in direct contact with the sample, producing an image of radioactivity distribution. In traditional autoradiography, silver halide film is commonly used resulting in resolution around 10 μm, influenced by emulsion thickness, silver halide grain size, and the strength of the development process [371]. In modern AR, predominantly solid-state detectors such as phosphor imaging plates, charge-coupled devices (CCD) and CMOS sensors are used. Phosphor imaging, using europium-doped barium fluorohalide crystals, offers higher sensitivity, reading latent images with laser stimulation and a photomultiplier tube. Resolution, still impacted by emulsion and grain properties, remains a limitation factor. CCD and CMOS sensors directly convert light into electrical signals; CCDs offer high sensitivity and low noise, while CMOS devices enable faster, lower-power, real-time imaging [372].

**Figure 30:**
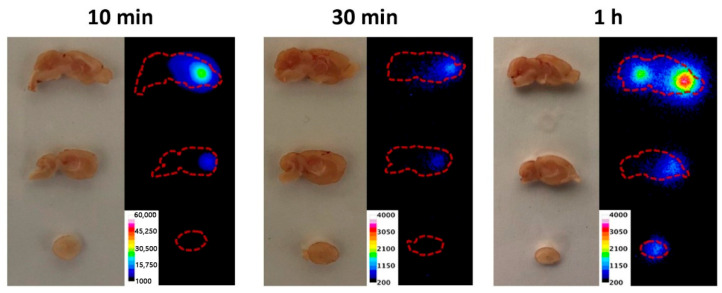
**Autoradiography imaging of mouse brain.** Autoradiography distribution images of AuNR radiolabeled with ^111^In in mouse brain following intranasal administration and sacrifice at 10 min, 30 min, or 1 h post-administration. Reproduced from [376], CC BY 4.0.

AR is applicable to a wide range of nanoparticles provided that they are labeled with radioactive isotopes. Radionuclides in nanoparticles can be a main component, encapsulated inside, or attached to the surface by direct conjugation or chelation [373]. Commonly used radionuclides include ^99m^Tc [374,375], ^111^In [376,377], ^131^I [378], and ^89^Zr [379,380,381] and many others depending on the required emission type and half-life. Interestingly, in one study by Han et al., gold nanorods (AuNR) radiolabeled with ^111^In were intranasal administered for brain targeting, with subsequent confirmation by autoradiography and other methods. AR revealed that AuNRs rapidly entered the brain, specifically the olfactory bulb (Figure 30), providing more detailed spatial information than SPECT/CT due to its superior sensitivity for detecting the low levels of AuNRs that reached the brain [376].

Another notable variant of this method is quantitative whole-body autoradiography (QWBA), which enables both visualization and absolute quantification of radiotracer distribution across the entire body [382,383]. In this method, the animal is euthanized, rapidly frozen, embedded, and cryosectioned into thin slices that are exposed on a phosphor imaging plate together with calibration standards. For instance, Al-Sid-Cheikh et al. used QWBA to compare the biodistribution of radiolabeled silver nanoparticles (^110m^Ag NP) and dissolved silver (^110m^AgNO_3_) in fish after dietary, waterborne, or intraperitoneal exposure [384]. QWBA revealed distinct patterns: AgNP localized mainly in peritoneal cavity, gut, and liver, while dissolved silver was more widely distributed, including kidney and gills. The method enabled detection of low-level tissue accumulations not observable with other methods.

AR provides quantitative, high-resolution data on nanoparticle distribution with high sensitivity. However, it is an ex vivo technique; therefore, tissue samples must be collected and processed, making real-time monitoring impossible. Additional limitations include radiation safety concerns, the short half-life of some isotopes, and the need for specialized handling and disposal protocols. In preclinical nanomedicine research, autoradiography is used less frequently compared to PET or SPECT, which provide real-time, whole-body, three-dimensional imaging of radiolabeled nanoparticles in living animals [385]. Nevertheless, AR is still used to verify results of in vivo imaging data as it is an ex vivo imaging method for quantitatively mapping the microscopic distribution of radionuclides in thin tissue sections (typically ~10 µm) with high spatial resolution (~1–100 µm). Despite its limitations, AR remains a valuable technique that provides essential data for validating nanomedicine biodistribution.

### 3.6. Local Biochemical and Histological Methods

#### 3.6.1. Histology with Staining

Histology is the gold standard for tissue examination in clinical pathology and life-science research; this method allows examining cells and tissues under a microscope (electron or light microscope) through staining and sectioning. Histological staining is widely applied in nanoparticle detection [386].

Two primary techniques are used to prepare histological sections for studying nanoparticle biodistribution: paraffin embedding and cryofixation. In the paraffin-based method, tissues are fixed in formalin, dehydrated through graded alcohols, and cleared in organic solvents such as xylene [387]. However, these procedures can disrupt or damage delicate nanostructures such as liposomes, micelles, and other lipid-based particles. For such nanoparticles, cryofixation followed by cryosectioning is the preferred approach [386].

**Figure 31:**
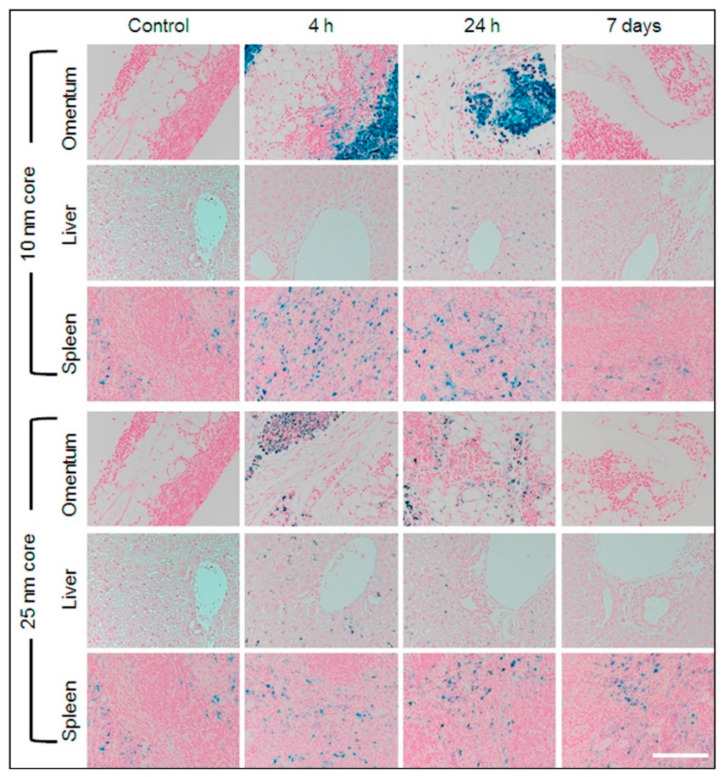
**Histology for NP’ distribution analysis.** Prussian blue iron staining of 10 nm s-SPION. Scale bar, 200 µm. Reproduced from [389], CC BY 4.0.

Bright-field microscopy can only resolve nanoparticles larger than 200 nm due to resolution limits. Thus, standard histology methods typically detect only nanoparticle clusters or aggregates. Specialized histochemical stains enable visualization of certain nanoparticles. Iron-based nanoparticles can be identified using Turnbull’s blue and Prussian blue (Figure 31) [388,389,390,391,392]. Alcian blue detects negatively charged sulfate groups, for example, Dendritic polyglycerol sulfate nanoparticles carry these groups in their shells [393]. Single-walled carbon nanotubes were visualized through covalent binding to gold nanoparticles with silver enhancement for improved detection [394]. Silver deposition not only increases contrast but also enlarges particle size, enabling sub-resolution gold nanoparticle localization [395]. Nanoparticles labeled with fluorescent dyes can be tracked in histological sections using fluorescence microscopy [396,397,398,399]. Many fluorescent dyes are available with several modifications, such as amino, carboxylic acid, maleimide and hydroxysuccinimide groups [400]. These functional groups are generally applied for the conjugation of peptides and proteins, including antibodies [401]. Quantum dots are light-emitting nanoparticles therefore they can be visualized by fluorescence microscopy without dyes [402,403].

Histology provides a cost-effective method for contextual evaluation of NP-induced pathological morphology without requiring ionizing radiation or contrast agents. However, light and fluorescence microscopy offer inherently limited resolution, preventing direct imaging of individual NP below ~200 nm and typically detecting only aggregates or labeled particles. This approach analyzes only select tissue sections (5–50 μm thick), potentially missing critical distributions. The technique is further constrained by its labor-intensive nature, susceptibility to human error during slide preparation/analysis, and challenges in specific cell-type identification—particularly for organic NP due to their biomimetic properties. Regarding fluorescence tracking, NP labeling with fluorophores may alter physicochemical behavior in vivo [404,405], while photobleaching during administration or tissue processing diminishes signal intensity. Tissue autofluorescence also necessitates appropriate control groups to resolve target signals [386,406].

#### 3.6.2. Immunohistochemistry

Immunohistochemistry (IHC) leverages antibody–antigen interactions to detect specific biomarkers within tissue sections, enabling precise localization of functionalized nanoparticles carrying antibodies, peptides, or other targeting molecules. This technique combines histological context with molecular specificity, making it invaluable for studying NP biodistribution, cellular uptake, and targeted delivery in complex biological environments. IHC can be applied both as a complement to standard histological staining and as a standalone method. The core procedure involves binding primary antibodies to surface molecules on nanoparticles or tissue, with visualization achieved through enzyme-conjugated (e.g., horseradish peroxidase, alkaline phosphatase) or fluorescent secondary antibodies and chromogenic/fluorescent substrates.

This method is particularly valuable for assessing tissue responses to NP as it visualizes specific cell types, activation states, and apoptotic or degenerative changes [407,408,409,410]. Direct visualization of nanoparticles using IHC is relatively rare. However, it has been successfully applied to detect cancer-derived exosomes using anti-CD63 IgG antibodies, achieving 20–50 nm resolution through super-resolution PALM/STORM imaging [411]. IHC also can be combined with Surface-enhanced Raman scattering [412].

While IHC offers exceptional specificity through antibody targeting of unique NP surface markers and enables ultra-high-resolution detection of small clusters when combined with advanced techniques, it requires accessible surface antigens and commercially available antibodies. Limitations include high reagent costs, antibody instability, potential for nonspecific interactions of antibodies with NP cores and aggregates.

#### 3.6.3. Western Blot/ELISA

Western blotting and enzyme-linked immunosorbent assay (ELISA) are powerful techniques leveraging antibody–antigen interactions to detect specific proteins associated with nanoparticles in biological samples. Western blotting separates proteins via gel electrophoresis, transfers them to a membrane, and detects targets using primary antibodies and enzyme-conjugated secondary antibodies (e.g., HRP or AP). This method enables assessment of NP-induced protein expression changes, such as altered KIF11 and MYC levels following siRNA-loaded NP treatment [409], detection of apoptotic proteins after NP-mediated drug delivery [413] and confirmation of SPAK knockdown by siRNA NP [414], RVG-targeted nanoparticle-mediated knockdown of caspase 3 [404]. The lower limit of Western blot detection is 0.1–1 ng of protein.

ELISA employs immobilized antigens in 96-well plates, which are detected through enzyme-linked antibodies with colorimetric/fluorometric readouts. It quantifies systemic distribution of NP-targeted proteins (e.g., cytokines, receptors) in blood or tissue homogenates, as demonstrated in studies of NFκB pathway activation by gold NP [408]. ELISA is highly sensitive and can detect proteins at concentrations ranging from 0.1 to 10 pg/mL.

Both techniques offer high sensitivity and specificity for confirming protein-NP associations. However, they require prior knowledge of target biomarkers and cannot directly visualize untagged NP in situ. Western blotting may miss low-abundance proteins due to transfer inefficiencies, while ELISA lacks spatial resolution. Rigorous controls and antibody optimization are essential to prevent false results and minimize nonspecific binding. The optimal strategy employs ELISA and Western blotting in combination with complementary nanoparticle detection methods, such as histochemical or immunohistochemical staining—to determine precise spatial localization of nanoparticles [407,414,415].

#### 3.6.4. RT-qPCR/In Situ Hybridization

RT-qPCR quantifies the mRNA levels of specific genes, providing valuable information about the impact of nanoparticles on gene expression in vivo. For example, when nanoparticles are used as delivery systems for small interfering RNAs (siRNAs) or messenger RNAs (mRNAs), RT-qPCR can assess the success of gene silencing or overexpression [404,409,414]. This technique is highly sensitive and allows the detection of as few as 1–10 copies of mRNA. However, while this method enables the assessment of the impact of nanoparticles on cells, it does not allow for the direct visualization of the nanoparticles themselves.

**Figure 32:**
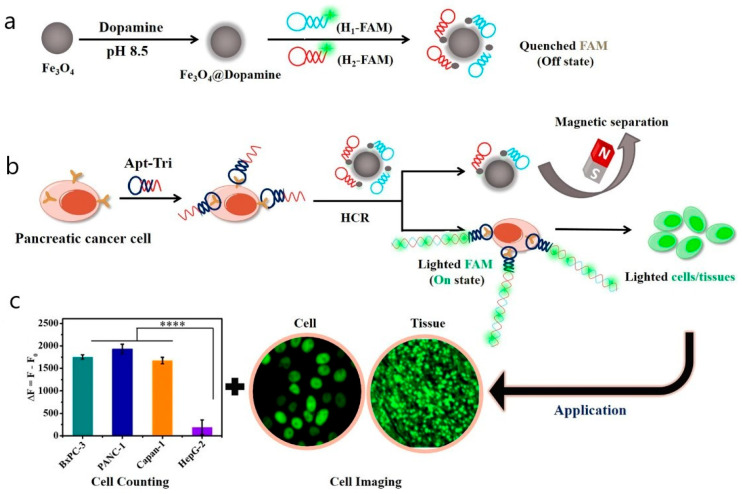
**Schematic illustration for detection of pancreatic cancer cells with FISH.** (**a**) The preparation process of the dopamine coated Fe_3_O_4_ nanoparticles (Fe_3_O_4_@DOP) and the attachment of H_1_-FAM (6-carboxyfluorescein) and H_2_-FAM onto the Fe_3_O_4_@DOP NP. (**b**) Diagram illustration for sensitive detection of pancreatic cancer cells based on Fe_3_O_4_@DOP NP and hybridization chain reaction amplification. (**c**) The application of the strategy in cell counting and imaging. **** *p* < 0.0001. Reproduced from [416], CC BY 4.0.

In contrast, in situ hybridization (ISH) provides spatial information by enabling the localization of nucleic acids (e.g., mRNA, microRNA, DNA) within tissue sections. This method involves hybridizing a labeled probe (fluorescent or radioactive) to the target nucleic acid sequence in the tissue, followed by microscopic analysis to determine its localization within cells and tissues. Fluorescence in situ hybridization (FISH) is a unique tool for the direct visualization of nanoparticles in cells or tissues (Figure 32) [416,417,418,419]. The resolution of this method is limited only by the resolving power of the microscope and it can reach 20–50 nm when using super-resolution microscopy methods—such as single-molecule FISH [417]. Also chromogenic in situ hybridization (CISH), in which the DNA probe is detected using a simple IHC-like peroxidase reaction, can be used in NP examination and give similar results as FISH and IHC [420].

Both RT-qPCR and in situ hybridization are highly sensitive techniques for detecting low-abundance nucleic acids in biological samples. However, RT-qPCR requires tissue homogenization, which eliminates spatial context. In situ hybridization preserves spatial information but is technically demanding and time-consuming, with risks of non-specific binding and increased cost due to the need for highly specific probes.

## 4. Discussion

Detecting nanoparticles in living systems can be compared to navigating unfamiliar terrain, and each imaging modality resembles a different navigation tool. In this analogy, fluorescence imaging is like a flashlight: it provides quick, sensitive glimpses of nearby structures, but its reach fades rapidly with depth. PET acts like GPS navigation: it offers a global and quantitative overview, yet depends on heavy infrastructure and specialized signals. MRI resembles a high-resolution satellite map: it delivers detailed structural information across the whole landscape, but at significant cost and with slower acquisition. Invasive assays are closer to digging soil samples: they provide ground-truth data, but only at the price of destroying part of the system. None of these tools alone is sufficient; only by combining them can the traveler move with confidence and precision. Likewise, no single imaging modality offers a “perfect” view of nanoparticle fate in vivo, and meaningful progress depends on integration. Every modality comes with trade-offs: between sensitivity and depth, between resolution and safety, between technical sophistication and practical accessibility. Ultimately, these trade-offs are dictated by physics itself: light scatters and is absorbed, magnetic fields decay, radioisotopes expose tissue to radiation. Recognizing these limitations is essential to choosing the right tool for the right biological question (Table 1).

Fluorescence imaging illustrates this tension better than any other modality. It is fast, sensitive, and widely used, but fluorescence intensity is often misinterpreted as a direct measure of how much of the injected dose (%ID) reached a given organ. In reality, scattering and absorption vary dramatically across tissues: a nanoparticle in the liver, spleen, or lung may produce very different signal intensities even if present in the same absolute amount. This makes %ID calculations based on raw fluorescence inherently unreliable. More meaningful measures, such as the organ accumulation factors, avoid these pitfalls by enabling relative comparisons within the same experiment. That said, fluorescence remains invaluable when speed matters. For rapid screening of dozens of nanoparticle designs, or for identifying promising formulations such as HER2-targeted PLGA carriers or ICG-loaded liposomes, nothing matches the throughput and accessibility of fluorescence-based assays.

This example highlights a broader rule: the physics of each technique defines its proper place. Optical methods, such as fluorescence, photoacoustics, SERS are superbly sensitive but shallow, limited to millimeters or a few centimeters. Nuclear approaches (PET, SPECT, Cherenkov imaging) can map nanoparticle distribution throughout the entire body with quantitative precision, but they require radiolabels and specialized facilities. Magnetic and acoustic techniques, such as MRI, MPQ, or ultrasound, strike a middle ground: they are safe for repeated monitoring, but less sensitive than nuclear or optical counterparts. No method is universally best, the challenge is to match the tool to the biological system and the clinical question.

Safety is another decisive factor. Imaging labels are not neutral passengers: they can shape the very biology they are meant to measure. Radiotracers bring radiation burden and regulatory hurdles. Quantum dots and other heavy-metal–based probes may persist in the liver and raise long-term toxicity concerns. Even iron oxide nanoparticles, which are generally considered safe, require careful coating strategies to prevent aggregation or rapid clearance by macrophages. In practice, the toxicological profile of a label often decides whether a method can move beyond the laboratory.

The balance between invasive and non-invasive approaches follows a similar logic. Invasive assays, such as electron microscopy, histology, mass spectrometry are irreplaceable when the goal is mechanistic insight: understanding how nanoparticles cross a membrane, where they accumulate inside a cell, or how they interact with organelles. But they come at the cost of destroying the sample. At later stages, when the goal is to follow nanoparticles across whole organs or organisms, non-invasive imaging becomes indispensable. MRI, PET, CT, ultrasound, and NIR imaging allow repeated measurements in the same living subject, capturing pharmacokinetics and biodistribution in real time. In practice, invasive and non-invasive methods complement one another: the first validate mechanisms, the second carry those insights into translational contexts.

This complementarity explains why hybrid and multimodal systems are gaining so much attention. PET/MRI, photoacoustic imaging with magnetic nanoparticles, and bioluminescence coupled with NIR probes all illustrate the power of combining modalities. By layering sensitivity, anatomical resolution, and quantitative accuracy, multimodal probes overcome the blind spots of single methods. Furthermore, advances in personalized medicine, targeted delivery, and theranostics are driving the development of nanoparticles with inherent multifunctionality, for instance, combining diagnostic and therapeutic capabilities or enabling combination therapies. These functionalities are realized by integrating compounds of diverse nature within a single nanostructure. Nanoparticles carrying multiple labels, such as radioactive, optical, magnetic, are not science fiction anymore; they are already being tested in preclinical and even early clinical studies. However, this progress introduces a significant challenge: the simultaneous detection of multimodal structures, which often necessitates the use of hybrid methodologies. Successful implementations of this principle include platforms like PET/CT, mass cytometry (a fusion of flow cytometry and ICP-MS), and the combination of LA-ICP-MS with histology [195,300,334]. Given that the current arsenal of such techniques remains limited, their further development constitutes a highly promising and actively evolving field. For instance, combining MRI with MPQ would provide both precise quantitative data on nanoparticle distribution and bioimaging of the process, while integrating MRI with SWIR would enable the simultaneous detection of both magnetic and fluorescent labels. Such synergistic approaches are crucial for a holistic, rather than fragmented, understanding of biological processes. Thus, a dynamic interplay between disciplines is created: the accelerating pace of discovery in biology generates a constant demand for novel analytical capabilities. This demand, in turn, drives innovation in physics and engineering, catalyzing the development of sophisticated, integrated tools needed to address the next generation of biological questions.

Our comparative analysis also makes one thing clear: not all nanoparticles are equally visible to all methods. Metallic particles (Au, Bi) are excellent for X-ray and plasmonic techniques but raise safety issues. Lipid and polymeric carriers are safer and clinically validated as drug delivery platforms, but need added labels and often yield weaker contrast. Magnetic nanoparticles are unmatched for MRI and MPQ, yet remain invisible to optical methods without functionalization. The choice of detection method is therefore inseparable from the nature of the nanoparticle itself.

Beyond physics and biology lies a very practical question: cost and infrastructure. PET and MRI deliver quantitative, clinically relevant data, but at the price of multimillion-dollar machines and highly trained staff. Fluorescence and NIR imaging, by contrast, are inexpensive, portable, and already used intraoperatively: for instance, in sentinel lymph node mapping during breast cancer or melanoma surgery. Specialized techniques such as LA-ICP-MS or ToF-SIMS offer unique, high-resolution insights, but their cost and complexity keep them confined to expert laboratories. Economics thus acts as a silent gatekeeper: only methods that balance performance with scalability can hope to achieve widespread clinical use.

Looking ahead, nanoparticle detection is likely to become a cornerstone of personalized medicine. Imaging will not just show where nanoparticles go: it will help determine whether therapy is working in a specific patient, and guide decisions about dosing, timing, and choice of formulation. Early glimpses of this future are already here: PET imaging with Cornell dots in oncology trials, intraoperative NIR fluorescence guiding tumor resections, SPION-enhanced MRI used to track inflammation. These examples show that translation is possible when sensitivity, safety, and practicality align.

The final question is: which methods could become gold standards in the coming decade? Likely winners will combine quantitative rigor (PET, SPECT, ICP-MS), repeatability and safety (MRI with SPIONs), and seamless integration with existing medical infrastructure. Optical methods will remain indispensable in discovery and preclinical research, gradually carving out niches in surgery and real-time monitoring. In the long run, the field will not be defined by a single technology, but by integration. Multimodal platforms, powered by artificial intelligence for image reconstruction and pharmacokinetic modeling, and guided by standardized protocols, are best positioned to make nanoparticle detection a routine part of clinical practice.

## 5. Conclusions

This review is intended as a practical handbook for newcomers to nanomedicine, summarizing how different detection strategies reveal complementary aspects of nanoparticle behavior in vivo. Fluorescence enables rapid, high-throughput screening but lacks depth and quantitative accuracy; PET provides reliable whole-body quantification but requires radiolabels and infrastructure; MRI offers detailed anatomical mapping at high cost; and invasive assays deliver ground-truth data at the expense of sample integrity. No single technique can capture the full picture—progress depends on combining modalities, matching them to specific biological questions, and acknowledging the trade-offs between sensitivity, resolution, safety, and accessibility.

The field now faces clear challenges: the absence of standardized protocols, the tendency to overinterpret optical data, and the economic and regulatory barriers to advanced imaging. At the same time, hybrid probes, AI-assisted analysis, and the integration of detection tools into personalized medicine provide exciting opportunities. Moving forward, the future of nanoparticle imaging will not be defined by a single “gold standard,” but by integrated toolkits that allow researchers and clinicians to design more rigorous experiments and, ultimately, to bring nanomedicine closer to routine clinical practice.

## Figures and Tables

**Table 1:** Comparative analysis of in vivo nanoparticle detection methods: main principles and applications.

№	Method	Principle of Detection	Detectable Nanoparticles	Limit of Detection	Resolution	Clinical Application	Application in Fundamental Science
1	Magnetic resonance imaging (MRI)	Enhanced signal of weighted images in presence of nanoparticles due to the effect on hydrogen proton relaxation	Nanoparticles based on gadolinium or iron oxides	In mM range	0.05–0.5 mm in clinic. 100 μm in animal experiments	FDA approved for imaging of blood vessels, lymph nodes, liver, and tumors (ferahema, resovist).	Organs and tumor imaging, angiography, molecular visualisation with functionalized NP
2	Magnetic Particle Quantification (MPQ)	Detection of non-linear response at combinatorial frequencies	Superparamagnetic nanoparticles, nanomaterials with nonlinear magnetic properties	60 zeptomoles or 0.33 ng of magnetic nanolabels	N/A	Not approved	NP-cells interaction in vitro, kinetics of NP circulation in vivo, biodistribution of NP ex vivo
3	Ultrasound imaging (USI)	Measurement of backscattered ultrasound waves intensity, which differs for tissues and cavities	Nanobubbles, gas vehicles, nanodroplets	Depends on nanoparticles structure and mechanism of action	1 mm	Ultrasound is used in clinic including microbubbles as contrast agents. Nanobubbles are in early clinical trials	Visualization of different structures including brain with nanoparticles as contrast agents
4	Photoacoustic imaging (PAI)	Detection of ultrasound waves created by the local pressure fluctuation after the conversion of absorbed light into heat	Metall and metall oxide nanoparticles, e.g., gold	In μM range	1–10 μm	The method is FDA approved. Nanoparticles are in preclinical studies	Tumor imaging with nanoparticles as contrast agents
5	Fluorescent molecular imaging	Measurement of emitted by fluorophore photons	Fluorescently labeled nanoparticles, quantum dots, anti-stokes nanoparticles and ruthenium nanoparticles	In nano- or picomolar range	1–2 mm with a penetration depth of up to 1.5 cm	Not approved	Nanoparticle biodistribution, tumor visualization with functionalized NP, lymphocytes path carting
6	Near infrared fluorescent imaging (NIR)	Measurement of emitted by fluorophore photons in transparency windows	Fluorescently labeled nanoparticles, quantum dots, anti-stokes nanoparticles and ruthenium nanoparticles	In nano- or picomolar range	50 μm with a penetration depth of up to 3 cm	FDA-approved for angiography and sentinel lymph node visualisation (ICG). Not approved for nanoparticle applications	Nanoparticle biodistribution, tumor and lymph nodes visualisation with functionalized NP
7	Light-sheet fluorescence microscopy (LSFM)	Measurement of emitted by fluorophore photons in near infrared or visible range	Fluorescently labeled nanoparticles, quantum dots, anti-stokes nanoparticles and ruthenium nanoparticles	In nano- or picomolar range	300–400 nm in lateral (x-y) plane. 1 μm in axial (z) plane	Not approved	Nanoparticle biodistribution, angiography
8	Surface-enhanced Raman scattering (SERS)	Nanostructure-mediated Raman signal enhancement	Gold, copper, platinum or silver nanoparticles functionalized with Raman reporter molecules	2.5 pM	10–20 nm	Not approved	Nanoparticle biodistribution, tumor visualization with functionalized NP
9	Photon correlation spectroscopy (PCS)	Measurement of the decay time of nanoparticle autocorrelation	PCS, XPCS: All types of commonly used nanoparticles. FCS: fluorescently labeled NP, quantum dots, anti-stokes nanoparticles and ruthenium nanoparticles	PCS and XPCS: nM range. FCS: nano- or picomolar range	Submicron resolution	FCS is used in clinic to detect amyloid β-protein aggregates in the cerebrospinal fluid of Alzheimer’s patients. Not approved for nano particle application	Measurement of NP size, aggregation and protein corona formation. FCS: measurement of blood vessels size and blood flow velocity.
10	Computed tomography (CT)	Measurement of attenuated X-ray radiation after absorption by nanoparticles	Nanoparticles with high atomic number (gold, bismuth, tantalum, liposomal or polymeric iodine nanoparticles)	Low sensitivity. Depends on atomic number and size of NP.	0.5–1 mm in clinic. 1–100 μm in preclinical trials	FDA approved for oncodiagnostic, angiography and stroke diagnosis. Preclinical trials for NP CT contrast agents	Bone structure visualization; organs and tumor imaging, angiography with contrast NP
11	Positron emission tomography (PET)	Detection of emitted positron	NPs, labeled with positron emitting radioisotopes	Nanomolar range	4 mm in clinic, 1 mm in preclinical studies	FDA-approved, but NPs yet in clinical trials	Tracking nanoparticle biodistribution and quantitative tumor accumulation evaluation
12	Single photon emission tomography (SPECT)	Detection of emitted single photon	NPs, labeled with single photon emitting radioisotopes	Nanomolar range	15 mm in clinic, 1 mm in preclinical studies	FDA-approved, including NPs use	Tracking nanoparticle biodistribution and quantitative tumor accumulation evaluation
13	Cherenkov luminescence imaging (CLI)	Detection of Vis/IR light emitted after radioactive decay	NPs, labeled with positron or electron emitting radioisotopes	Nanomolar range	1 mm, worth with depth	Undergoing clinical trials	Tracking nanoparticle biodistribution and hard, but possible quantitative tumor accumulation evaluation
14	X-ray fluorescence (XRF)	Detection of fluorescence, emitted by X-rays excited atoms	Nanoparticles of elements with Z > 23	50 mkg/mL in living mice	100 μm in living mice	Used in fundamental science	Study of biodistribution of NP
15	In vivo flow cytometry (IVFC)	Detection of nanoparticles on cells in the bloodstream or lymph flow	Fluorescently labeled, plasmonic, magnetic NPs	Single nanoparticles-labeled cell (typically, 3–10 nanoparticles per 1 cell)	Single-cell resolution. Temporal resolution 0.1–1 ms	Used in fundamental science	Real-time detection of nanoparticles interaction with circulating cells
16	Electron microscopy (EM)	Electrons probe the sample, producing an image	Any type of nanoparticles	Individual NP can be visualized	SEM: 1–10 nm. TEM: 0.1–0.2 nm	Electron microscopy used selectively in clinics without nanoparticles	Study of the size and shape of NP and their accumulation in tissues.
17	Confocal and fluorescence microscopy	Detection of fluorescent signal	Fluorescent nanoparticles and particles with fluorescent labels	Depends on the amount of fluorophore	From 180 to 800 nm. STED: from 20 to 40 nm	Approved without nanoparticles	Nanoparticle biodistribution, tumor visualisation with functionalized NP
18	Multiphoton microscopy	The probe interacts with two or three photons at once and fluorescence is detected	Quantum dots, upconversion nanoparticles, carbon dots, dye-labeled particles, plasmonic nanoparticles	Depends on fluorophore	subcellular resolution	Used in clinical trials	Nanoparticle-cell interaction in vitro, in vivo imaging
19	Fluorescence Lifetime Imaging Microscopy (FLIM)	Detecting photon emission lifetimes.	Fluorescent nanoparticles and particles with fluorescent labels	Depends on fluorophore	Matches the resolution of the fluorescence microscopy	Used in fundamental science	Study of cell metabolism and nanoparticle imaging
20	Atomic force microscopy (AFM)	measuring the forces between a sharp tip and a sample surface	Noble metal nanoparticles, quantum dot, single-walled carbon nanotubes	Depends on NP size	0.1 nm	Used in fundamental science	Study of the size and shape of NP and nanoparticle-cell interaction in vitro
21	Photothermal microscopy	Detection of photothermal signal after the absorption of light by a nanoparticle	Gold nanoparticles, carbon nanotubes, semiconductor quantum dots, polymer nanoparticles with photothermal absorbers	absorption cross-section as small as a few 10^−15^ cm^2^	50 nm	Used in fundamental science	Real-time monitoring of nanoparticle-cell interaction
22	Flow cytometry (FCM)	Measurement of forward/side light scattering and laser-induced fluorescence from single cells or particles in a high-speed laminar flow	Fluorescently labeled NPs; non-fluorescent NPs (Ag, Zn, Au, and others) via SSC	Depends on fluorophore brightness or NP size	Single-cell resolution	FDA-approved for hematology and immunology (ex vivo); not approved for nanoparticle applications	Nanoparticle-cell interaction studies, uptake quantification, targeting efficiency
23	Cytometry by time-of-flight (CyTOF)	Labeled cells are ionized and analyzed by mass-to-charge ratio	Metal nanoparticles or isotope-containing nanoparticles	Element-dependent with achievable sensitivity of 10 nanoparticles/cell	Single-cell resolution	Blood samples immune profiling in clinical trials	High-dimensional single-cell profiling, quantitative analysis of nanoparticle uptake by cells
24	Inductively coupled plasma mass spectrometry (ICP-MS)	Elemental analysis of the ionized sample based on mass-to-charge ratio	Metal nanoparticles or isotope-containing nanoparticles	From <1 to >100 ng/L, element-dependent	None	Study of the elemental composition of hair, blood, nails and urine, clinical trials of drugs	Industrial analytics, archaeological studies, environmental monitoring, biomedical research, and forensic science
25	Laser ablation ICP-MS (LA-ICP-MS)	Spatial resolved elemental analysis of the sample based on mass-to-charge ratio	Metal nanoparticles or isotope-containing nanoparticles	In the range from a few ng/g to µg/g and more	10–100 μm for nanosecond lasers and <1 μm for femtosecond lasers	Study of the drug distribution in clinical trials	Elemental mapping and bioimaging in biological specimens, including both animal and plant tissues
26	Time-of-Flight Secondary Ion Mass Spectrometry (ToF-SIMS)	Primary ions eject secondary ions, which are detected.	Any type of nanoparticles	up to tens of ppm	up to 100 nm	Not approved	For lipidomics, metabolomics, and nanoparticle biodistribution/toxicity.
27	Chemiluminescent analysis	Light emission generated by chemical reactions catalyzed by peroxidase enzymes or nanozymes	Noble metal NPs (Au, Ag, Pt), metal oxides (CuO, Fe_3_O_4_, Co_3_O_4_), carbon-based NPs or NPs labeled with enzyme/substrate	Nanomolar range	Limited by tissue light scattering	Not approved	Nanozyme research, biosensing, and in vivo imaging of oxidative microenvironments
28	Bioluminescent analysis	Enzymatic light emission	NPs conjugated with luciferase or substrate	LOD reported only for in vitro systems (picomolar to femtomolar)	<1 mm in vivo	Not approved	Tracking nanoparticle biodistribution
29	Autoradiography (AR)	Detection of ionizing radiation	NPs labeled with radioactive isotopes	No LOD explicitly reported; generally high sensitivity enabling detection of low-level tissue accumulations	High spatial resolution (~10 μm)	Not approved; used only in preclinical validation studies	Quantitative mapping of radiolabeled NP biodistribution in tissue sections, validation of in vivo imaging data, and in vivo imaging
30	Histology	Tissue staining and microscopy	Metallic, stainable, fluorescent-labeled, quantum dots	LOD not reported	~200 nm (light)	Perls prussian blue	Study of NPs biodistribution, tissue responses on treatment
31	Immunohistochemistry (IHC)	Antigen–antibody interaction detected with microscopy	NPs with proteins	Antibody-dependent (~pmol protein)	Down to 20–50 nm (super-resolution techniques)	Used in fundamental science	Study of NPs biodistribution, tissue responses on treatment
32	Western blot	Protein separation and labeling with antibodies	NPs with protein tags or inducing proteins	0.1–1 ng protein	N/A	Used in fundamental science	NP biological effects on protein expression
33	ELISA	Antigen immobilization and labeling with antibodies	NPs with protein tags or inducing proteins	0.1–10 pg/mL protein	N/A	Used in fundamental science	NP biological effects on protein expression
34	RT-qPCR	mRNA/DNA amplification	NPs affecting gene expression	1–10 mRNA copies	N/A	Used in fundamental science	NP biological effects on gene expression

## Data Availability

Data are contained within the manuscript.

## Abbreviations

The following abbreviations are used in this manuscript:

AFM	atomic force microscopy
AR	autoradiography
BL	bioluminescence
CCD	charge-coupled devices
CISH	chromogenic in situ hybridization
CL	chemiluminescence
CLI	Cherenkov Luminescence Imaging
CMOS	complementary metal-oxide semiconductor
CRET	Cherenkov radiation energy transfer
CT	computed tomography
CTC	circulating tumor cells
CyTOF	cytometry by time-of-flight
ELISA	enzyme-linked immunosorbent assay
EM	electron microscopy
FCM	flow cytometry
FDA	Food and Drugs Administration
FDG	fluorodeoxyglucose
FISH	fluorescence in situ hybridization
FITC	fluorescein isothiocyanate
FLIM	fluorescence lifetime imaging microscopy
FRET	Förster resonance energy transfer
FSC	forward scatter
FCS	fluorescence correlation spectroscopy
HER2	human epidermal growth factor receptor 2
ICP	inductively coupled plasma
ICP-MS	inductively coupled plasma mass spectrometry
ICP-TOF-MS	inductively coupled plasma time-of-flight mass spectrometry
ICG	indocyanine green
ID	injected dose
IHC	immunohistochemistry
ISH	in situ hybridization
IVFC	in vivo flow cytometry
LA-ICP-MS	laser ablation inductively coupled plasma mass spectrometry
LOD	limit of detection
LSFM	light sheet fluorescence microscopy
MMUS	magnetomotive ultrasound
MNP	magnetic nanoparticle
MPQ	magnetic particle quantification
MRI	magnetic resonance imaging
NIR	near-infrared
NP	nanoparticles
PAI	photoacoustic imaging
PALM	photoactivated localization microscopy
PAM	photoacoustic microscopy
PAT	photoacoustic tomography
PCS	photon correlation spectroscopy
PEG	polyethylene glycol
PET	positron emission tomography
PLGA	poly (lactic-co-glycolic) acid
SAIO	supramolecular amorphous-like iron oxide nanoparticles
SDD	silicon drift detector
SEM	scanning electron microscopy
SERS	surface-enhanced Raman scattering
SIM	structured illumination microscopy
siRNAs	small interfering RNAs
SP-ICP-MS	single-particle ICP-MS
SPECT	single photon emission computed tomography
SPION	superparamagnetic iron oxide nanoparticles
SSC	side scatter
STED	stimulated emission depletion
STORM	stochastic optical reconstruction microscopy
SWIR	short-wave infrared
TEM	transmission electron microscopy
ToF-SIMS	time-of-flight secondary ion mass spectrometry
USI	ultrasound imaging
XPCS	X-ray photon correlation spectroscopy
XRF	X-ray fluorescence
